# Supplementary figures and images for: CryoEM structures of the human CLC-2 voltage gated chloride channel reveal a ball and chain gating mechanism
Source: bioRxiv. 2023 Nov 29:2023.08.13.553136. Originally published 2023 Aug 15. Preprint. [Version 2] doi: 10.1101/2023.08.13.553136 (PMC10462068; doi:10.1101/2023.08.13.553136)

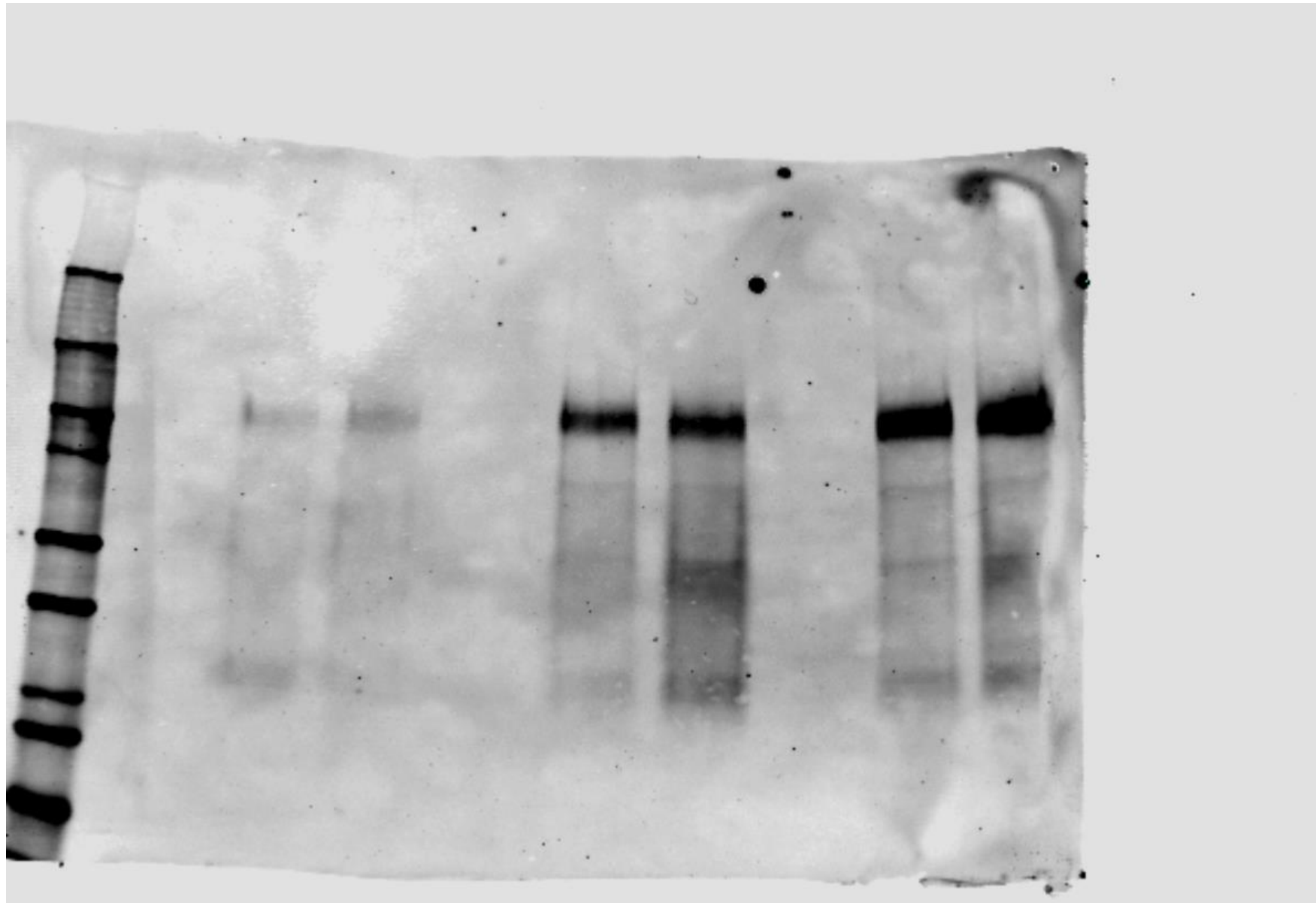

Supplement: Supplement 1 [file media-1.pdf]
