# Supplementary material for: CryoEM structures of the human CLC-2 voltage gated chloride channel reveal a ball and chain gating mechanism
Source: bioRxiv. 2023 Nov 29:2023.08.13.553136. Originally published 2023 Aug 15. Preprint. [Version 2] doi: 10.1101/2023.08.13.553136 (PMC10462068; doi:10.1101/2023.08.13.553136)
Supplement: Supplement 3 — Figure 2 - figure supplement 1. Micrograph and 2D classes and structure validation of CLC2-TM. (A) Representative motion-corrected cryo-EM micrograph. (B) 2D class averages. (C) Gold standard FSC plots calculated in cryoSPARC. (D) Local resolution of the cryo-EM map of the CLC2-TM. (E) Model validation using Q-scores (Pintilie et al., 2020) of subunit A (left) and subunit B (right). The black line represents the expected Q-score at respective resolution based on the correlation between Q-scores and map resolution. Figure 2 - figure supplement 2. CryoEM workflow of the CLC2 single-particle cryoEM data processing. A total of 14,198 movie stacks were collected on a 300 kV Titan Krios cryo-electron microscope. cryoSPRAC was used for 2D classification, and the CLC2-TM density map was obtained after 2D classification. Relion was used for 3D classification and yielded two conformations differing in the CTD: CLC2-CTDsym and CLC2-CTDasym. Resolutions shown refer to the whole protein molecule. Figure 2 - figure supplement 3. Helix map of CLC2-TM. cryo-EM densities and model of CLC-2 transmembrane helices. Figure 2 - figure supplement 4. Egate position and Cl− binding sites (Sext and Scen). (A) Comparison between CLC-2 (purple), CLC-1 (light blue), and cmCLC (salmon pink). Egate (E205 in CLC-2, E232 in CLC-1, and E210 in cmCLC), SerC (S162 in CLC2, S189 in CLC-1, and S165 in cmCLC) and TyrC (Y553 in CLC-2, Y578 in CLC-1, and Y515 in cmCLC) are shown as sticks. In CLC-2 and cmCLC, Sext is occupied by Cl−, and Scen is occupied by Egate. Egate is in the “down” position. In CLC-1, Sext is occupied by Cl−, and Scen lacks anion density. Egate is in the “out” position, away from the Cl−-permeation pathway. (B) Overlay view (stereo) of panel A. (C) Overlay with the CLC-2 Egate conformations modeled in PDB ID: 7XJA (Ma et al., 2023). Figure 3 - figure supplement 1. Cl− pathway in CLC-2 and CLC-1. (A) CLC-2 Cl− pathway as shown in Figure 3 but omitting sections with pore radius less tha [file NIHPP2023.08.13.553136v2-supplement-3.pdf]

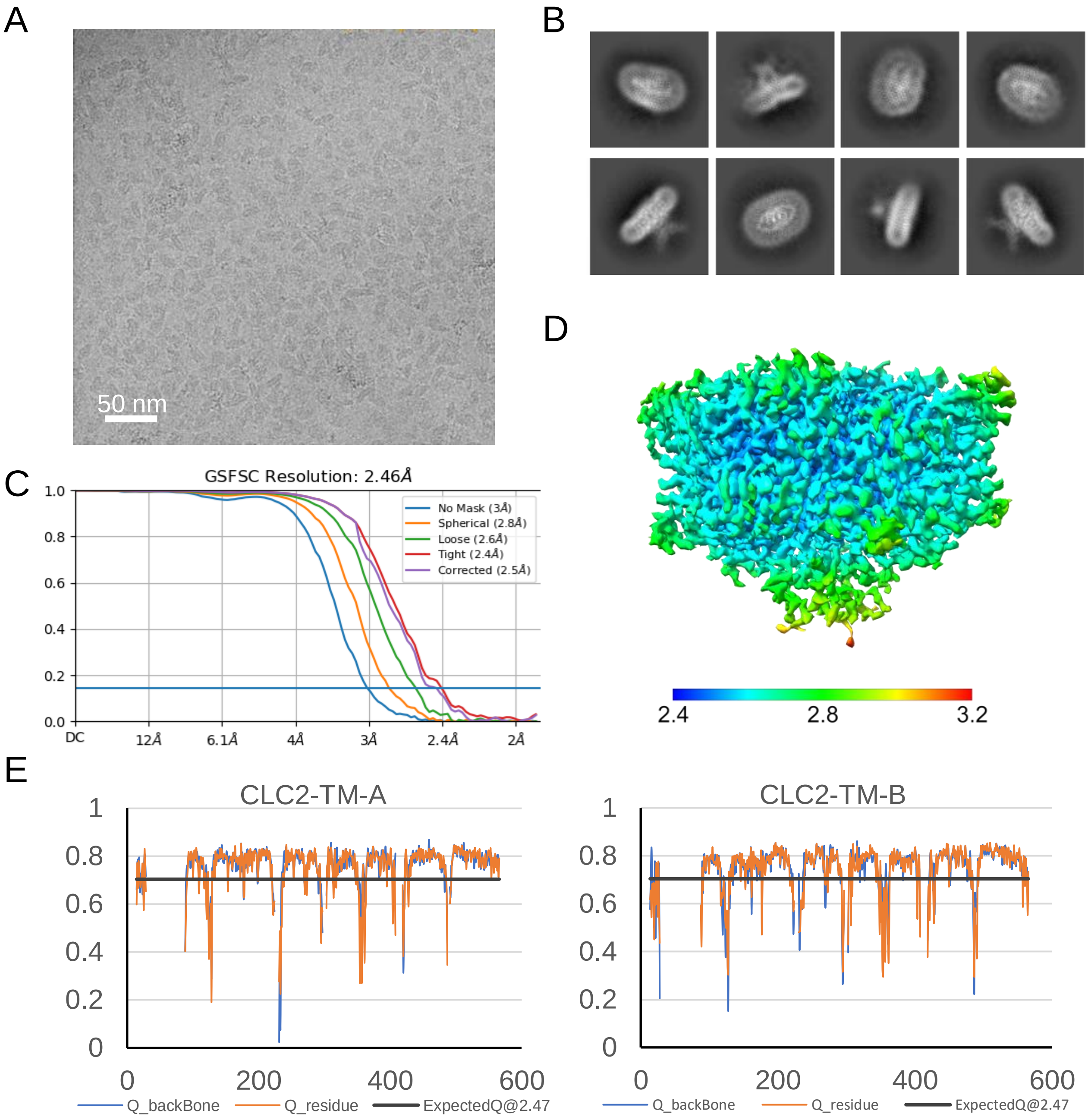

Figure 2 - figure supplement 1. Micrograph and 2D classes and structure validation of CLC2-TM. (**A**) Representative motion-corrected cryo-EM micrograph. (**B**) 2D class averages. (**C**) Gold standard FSC plots calculated in cryoSPARC. (**D**) Local resolution of the cryo-EM map of the CLC2-TM. (**E**) Model validation using Q-scores (Pintilie et al., 2020) of subunit A (left) and subunit B (right). The black line represents the expected Q-score at respective resolution based on the correlation between Q-scores and map resolution.

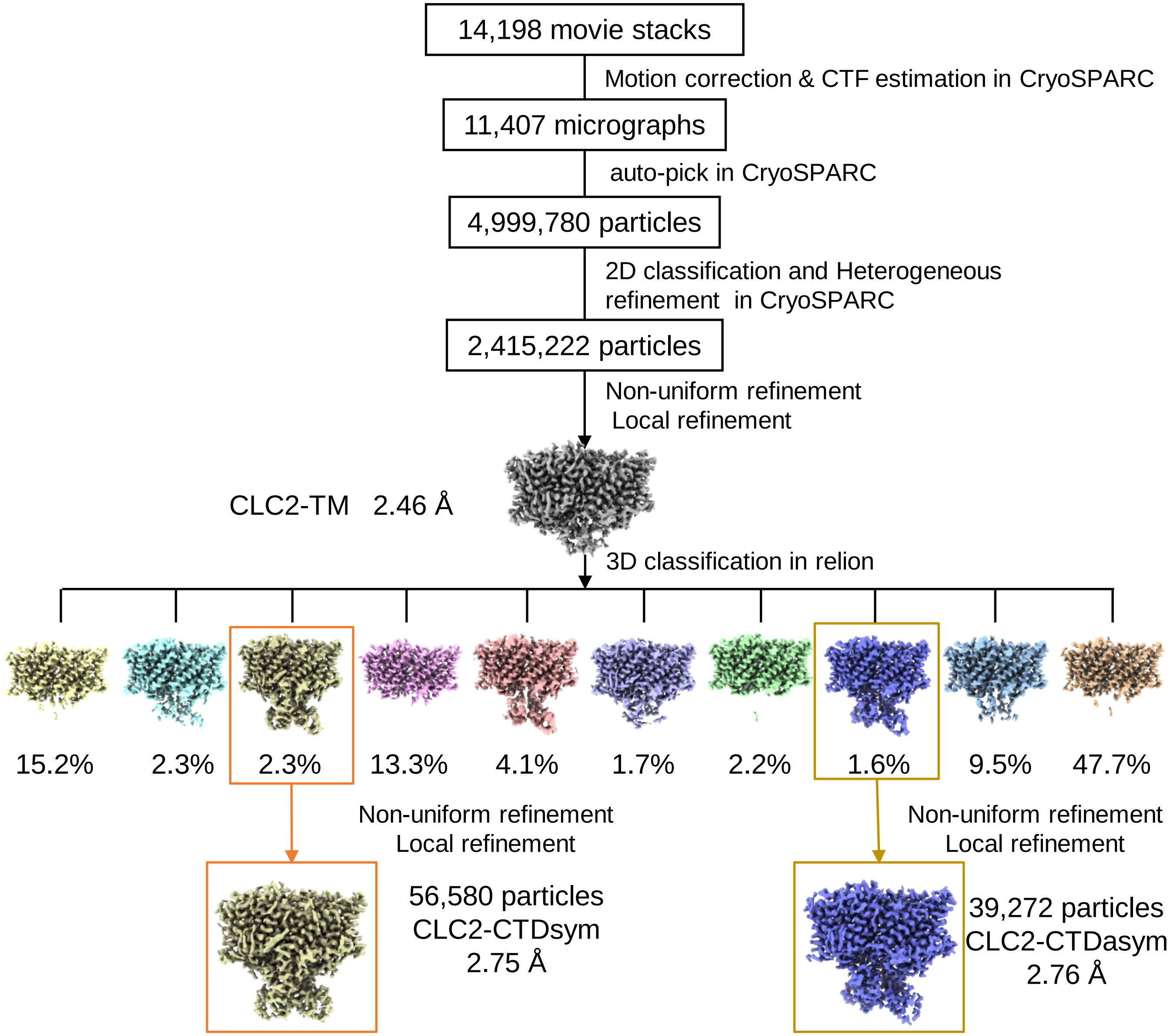

Figure 2 - figure supplement 2. CryoEM workflow of the CLC2 single-particle cryoEM data processing. A total of 14,198 movie stacks were collected on a 300 kV Titan Krios cryo-electron microscope. cryoSPRAC was used for 2D classification, and the CLC2-TM density map was obtained after 2D classification. Relion was used for 3D classification and yielded two conformations differing in the CTD: CLC2-CTDsym and CLC2-CTDasym. Resolutions shown refer to the whole protein molecule.

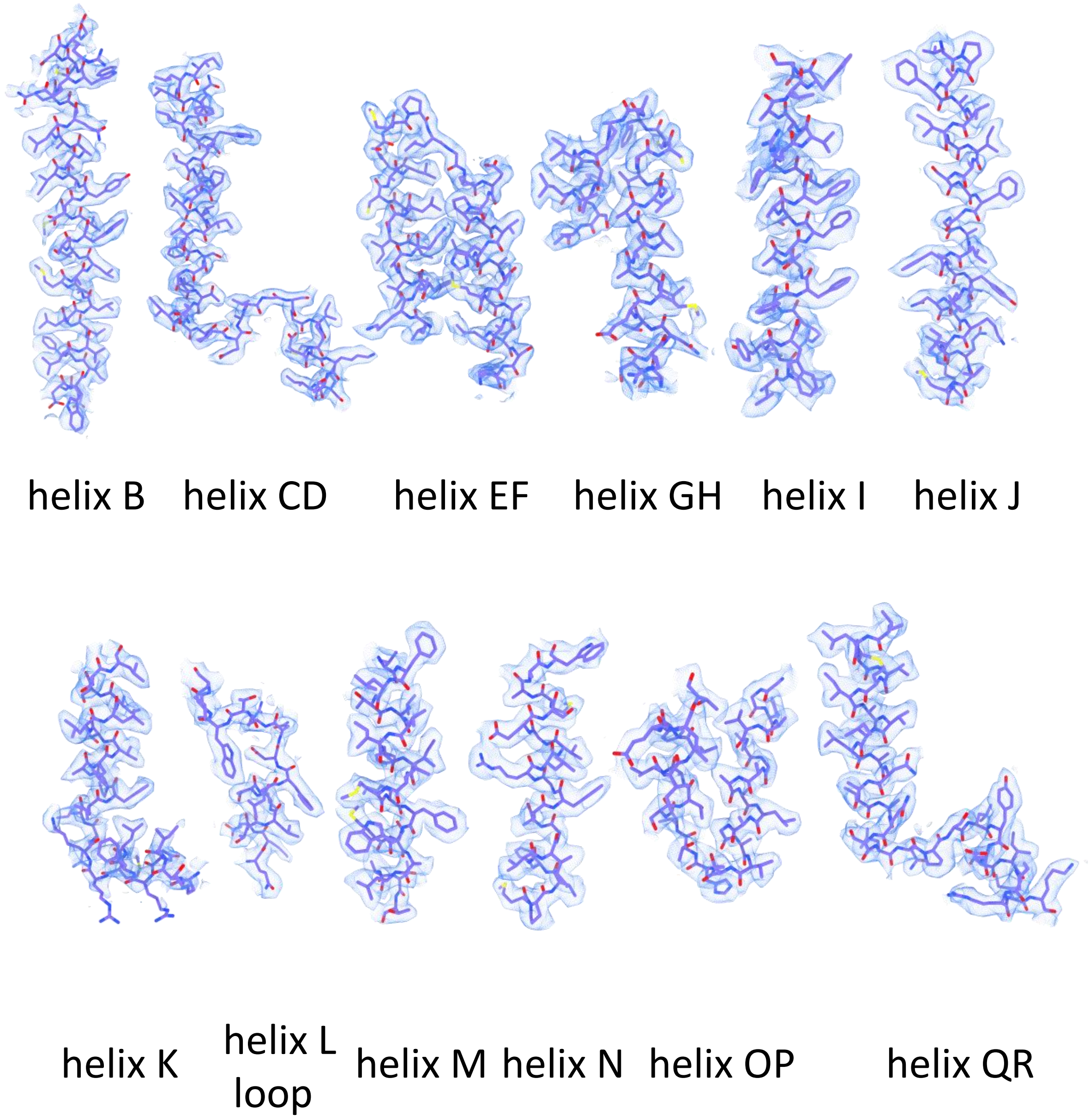

Figure 2 - figure supplement 3. Helix map of CLC2-TM. cryo-EM densities and model of CLC-2 transmembrane helices.

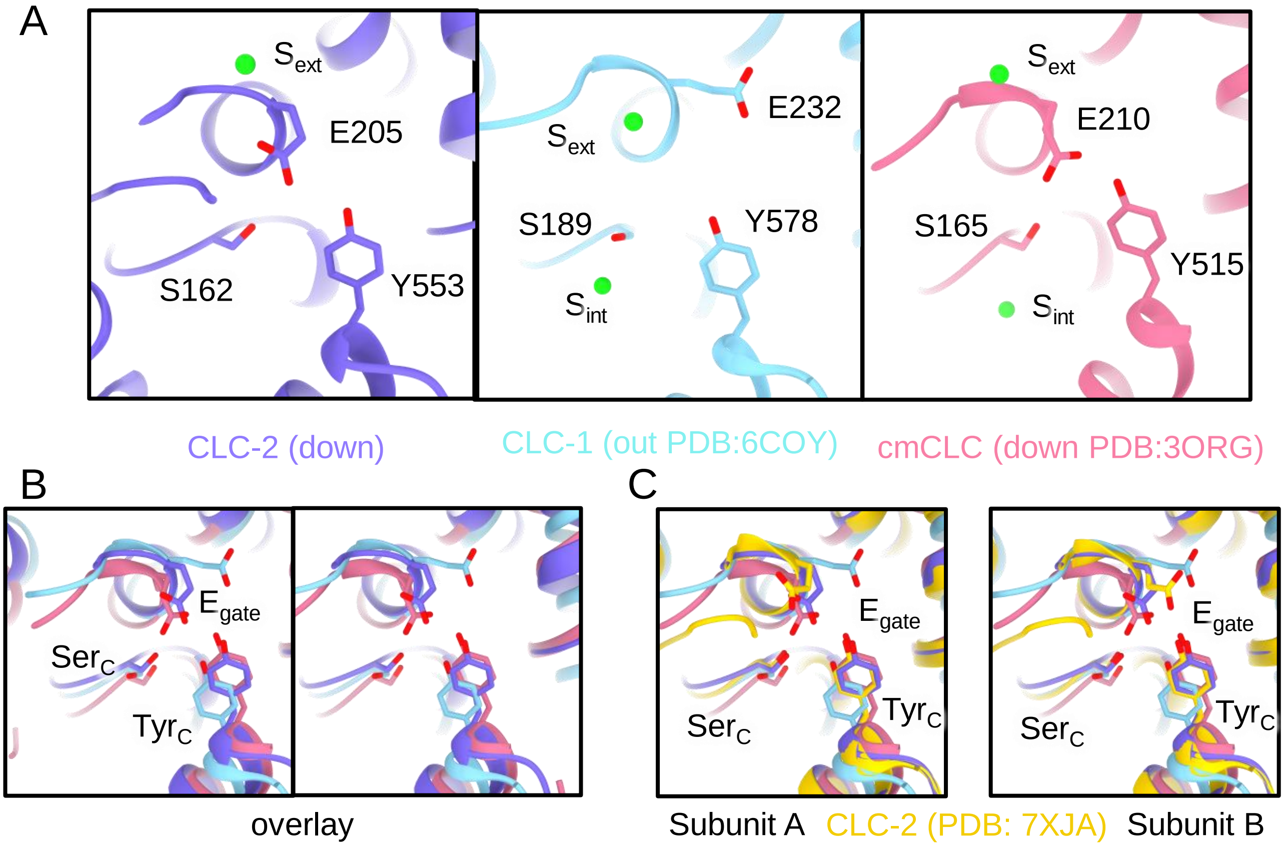

Figure 2 - figure supplement 4. E_{gate} position and Cl⁻ binding sites (S_{ext} and S_{cen}). (**A**) Comparison between CLC-2 (purple), CLC-1 (light blue), and cmCLC (salmon pink). E_{gate} (E205 in CLC-2, E232 in CLC-1, and E210 in cmCLC), Ser_C (S162 in CLC2, S189 in CLC-1, and S165 in cmCLC) and Tyr_C (Y553 in CLC-2, Y578 in CLC-1, and Y515 in cmCLC) are shown as sticks. In CLC-2 and cmCLC, S_{ext} is occupied by Cl⁻, and S_{cen} is occupied by E_{gate}. E_{gate} is in the "down" position. In CLC-1, S_{ext} is occupied by Cl⁻, and S_{cen} lacks anion density. E_{gate} is in the "out" position, away from the Cl⁻-permeation pathway. (**B**) Overlay view (stereo) of panel **A**. (**C**) Overlay with the CLC-2 E_{gate} conformations modeled in PDB ID: 7XJA (Ma et al., 2023).

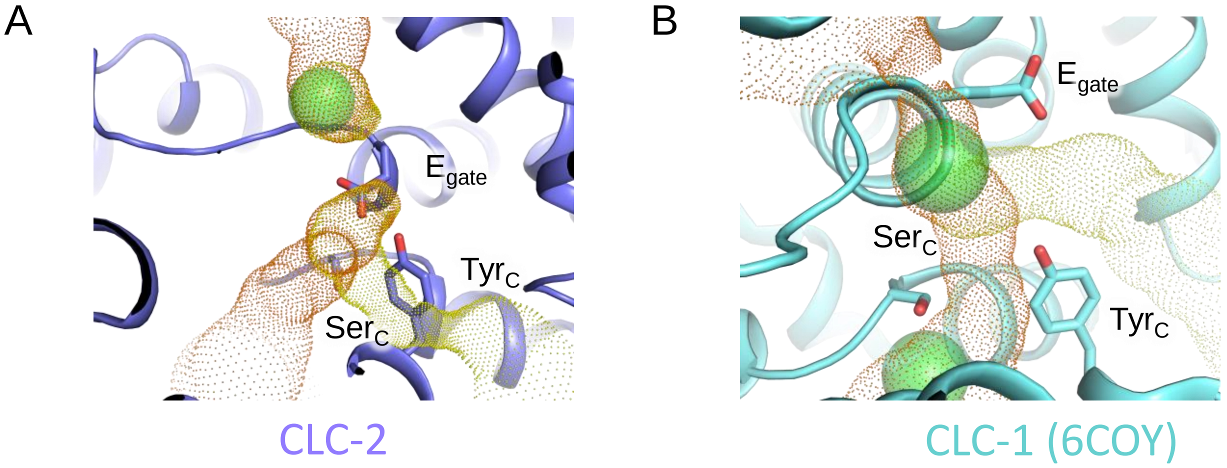

Figure 3 - figure supplement 1. Cl⁻ pathway in CLC-2 and CLC-1. (**A**) CLC-2 Cl⁻ pathway as shown in Figure 3 but omitting sections with pore radius less than 1Å. E_{gate}, occupying the S_{cen} site, blocks the canonical Cl⁻ pathway. (**B**) same as panel A, for CLC-1 (PDB ID:6coy).

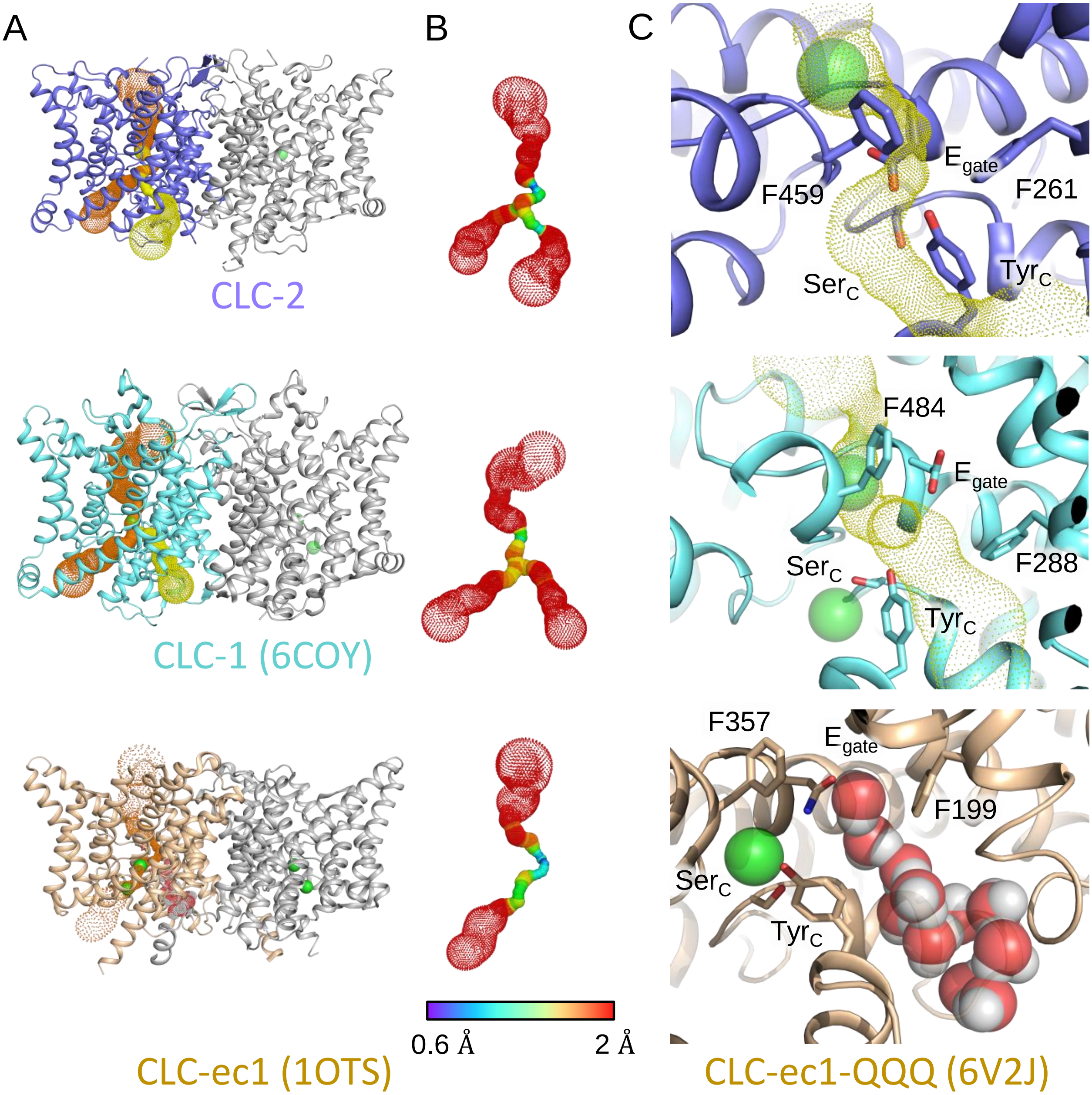

Figure 3 - figure supplement 2. Comparison of primary and secondary Cl⁻ pathways with transporter Cl⁻ and H⁺ pathways. (**A**) The top two panels are repeated from Figure 3A, indicating the primary (orange) and secondary (yellow) caver-detected pores in CLC-1 and CLC-2. The lower panel shows CLC-ec1 (PDB ID: 1OTS), a representative for the CLC transporters, where caver detects only the primary pore (orange dots as for CLC-1 and CLC-2). (**B**) Detected pore radii show a longer constriction in the CLC-ec1 transporter compared to the CLC-1 and CLC-2 channels. (**C**) Zoomed-in view of the secondary-pore region for CLC-2 (top), CLC-1 (middle) and CLC-ec1 (bottom). The CLC-ec1 panel shows water wires (space-filled) detected in simulations of the QQQ mutant structure (PDB ID: 6V2J) (Chavan et al., 2020).

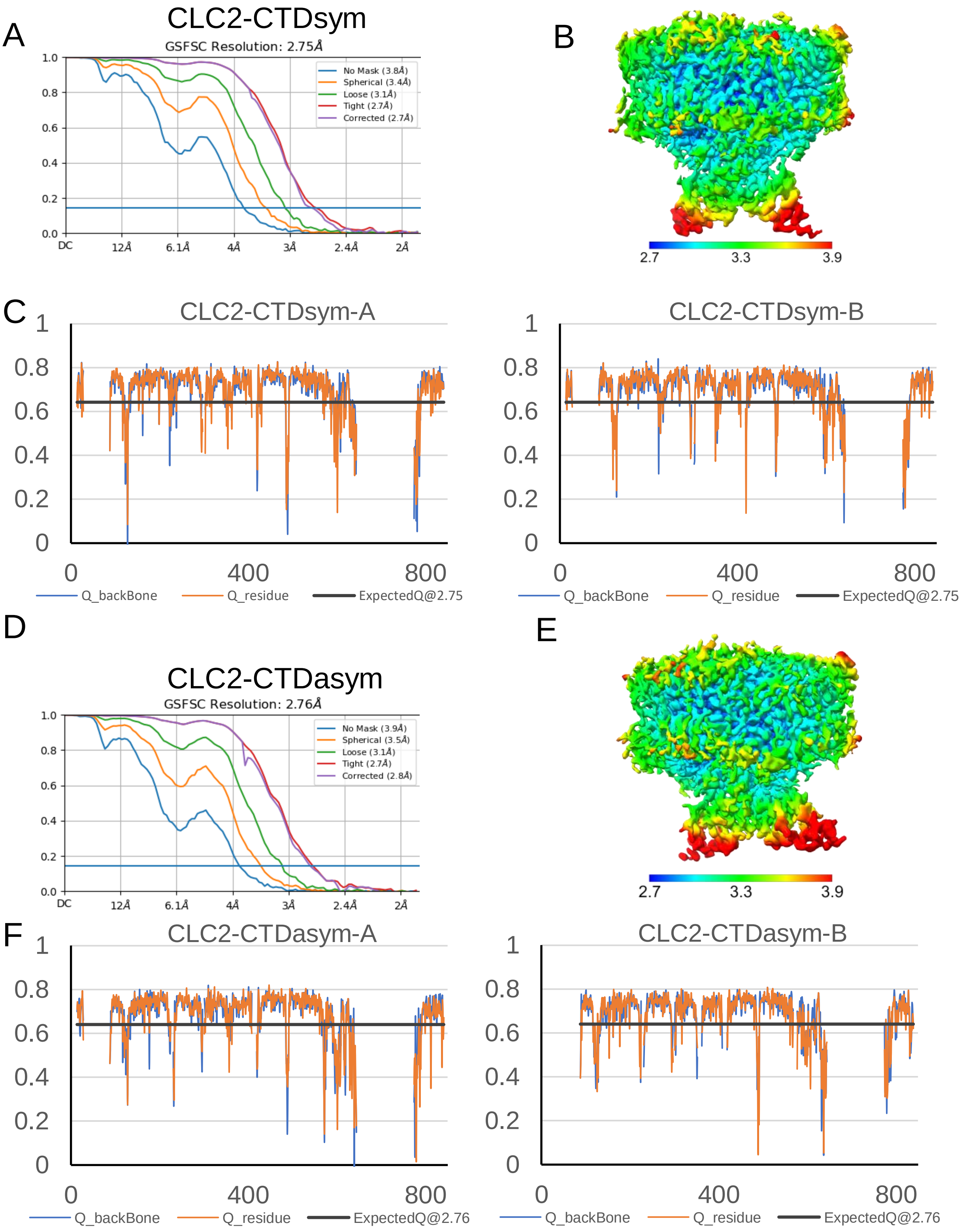

Figure 4 - figure supplement 1. Structure validation of CLC2-CTDsym and CLC2-CTDasym. (**A**) Gold standard FSC plots calculated in cryoSPARC for CLC2-CTDsym. (**B**) Local resolution of the cryo-EM map of the CLC2-CTDsym. (**C**) Model validation using Q-scores of subunit A (left) and subunit B (right) of CLC2-CTDsym. The black line represents the expected Q-score at respective resolution based on the correlation between Q-scores and map resolution. (**D-F**) the same as (**A-C**) for CLC2-CTDasym**.**

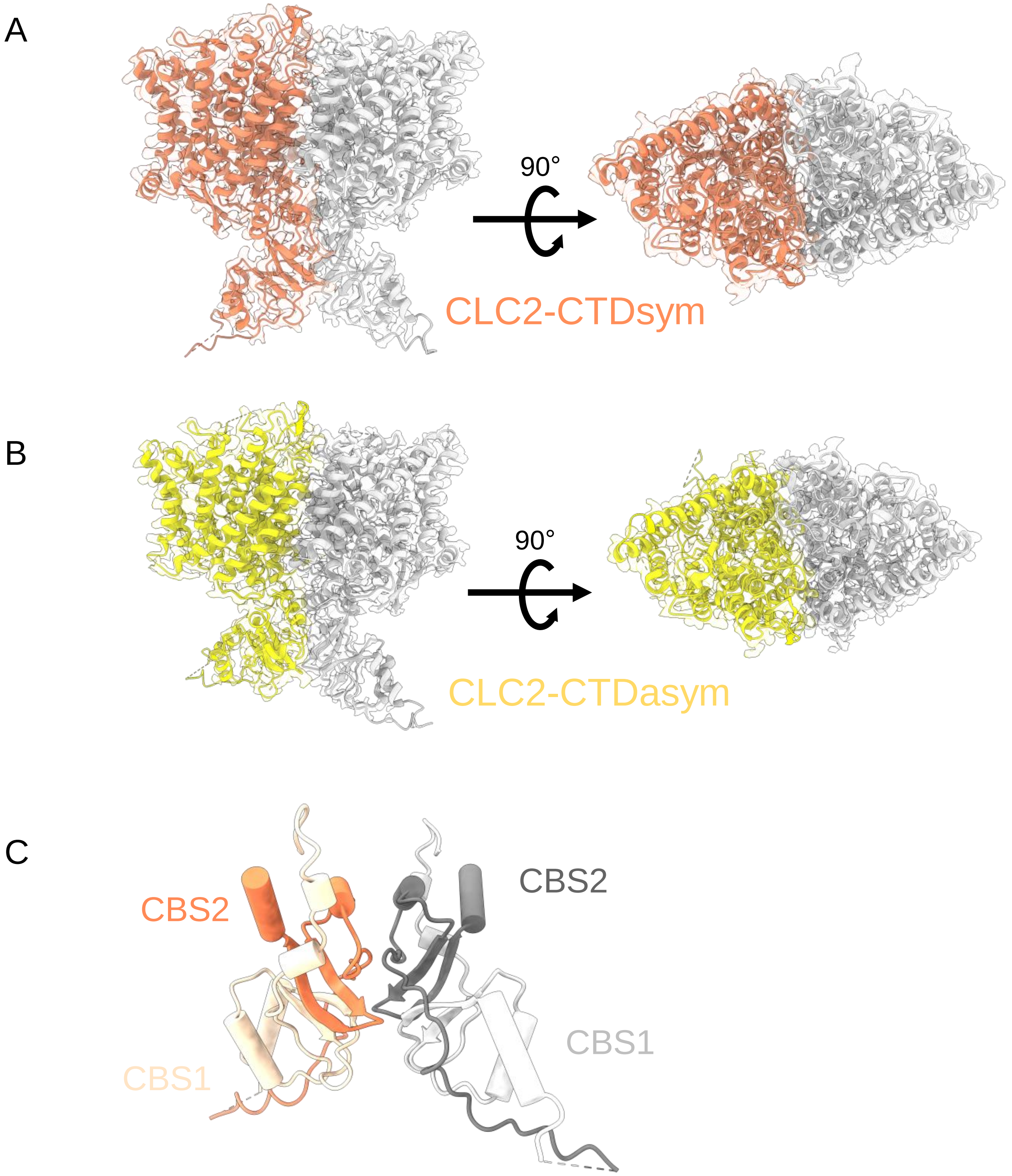

Figure 4 – figure supplement 2. CryoEM density maps for the two CTD-containing CLC-2 conformations, overlaid with structural models. (**A**) CLC2-CTDsym. (**B**) CLC2-CTDasym. Side view (left) and top view (right) are shown. (**C**) CTD of CLC2-CTDsym shows two CBS domains on each subunit (orange and light orange for subunit A; gray and light gray for subunit B).

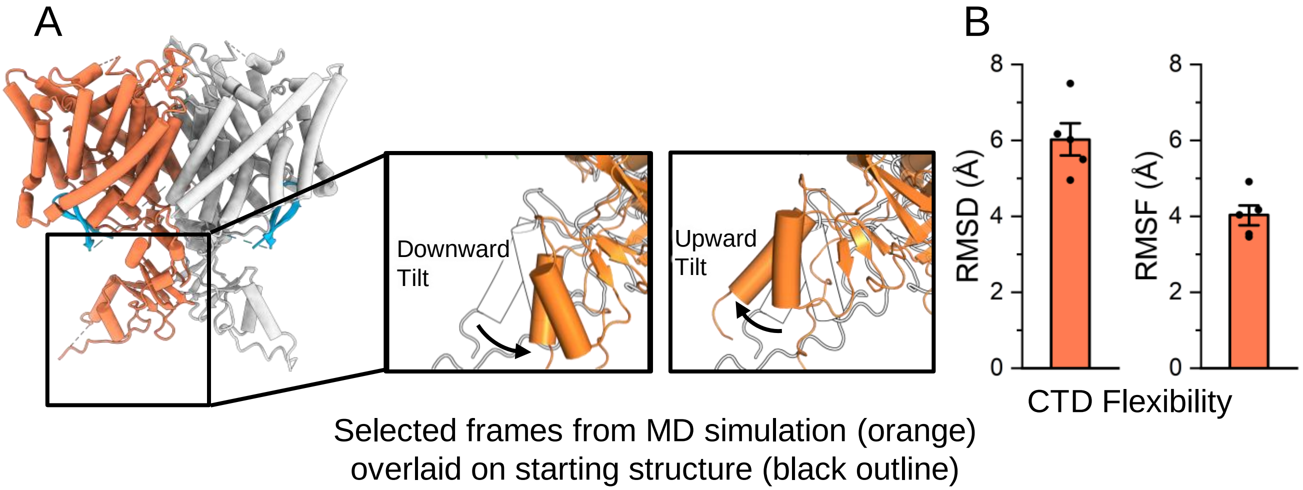

Figure 4 - figure supplement 3. MD analysis indicates conformational flexibility of the CTD. (**A**) The CTD is highly mobile relative to the transmembrane domain, and often tilts upward or downward relative to its initial location. In the inset images, two representative frames from simulation are shown (orange) overlaid on the starting structure (black outline). (**B**) The RMSD (root mean square deviation) and RMSF (root mean square fluctuation) of the cytoplasmic domain backbone were calculated after aligning frames on the transmembrane domains. Bars show the mean of five independent simulations, each 2.0 μs in length. Error bars are 68% confidence intervals of the mean.

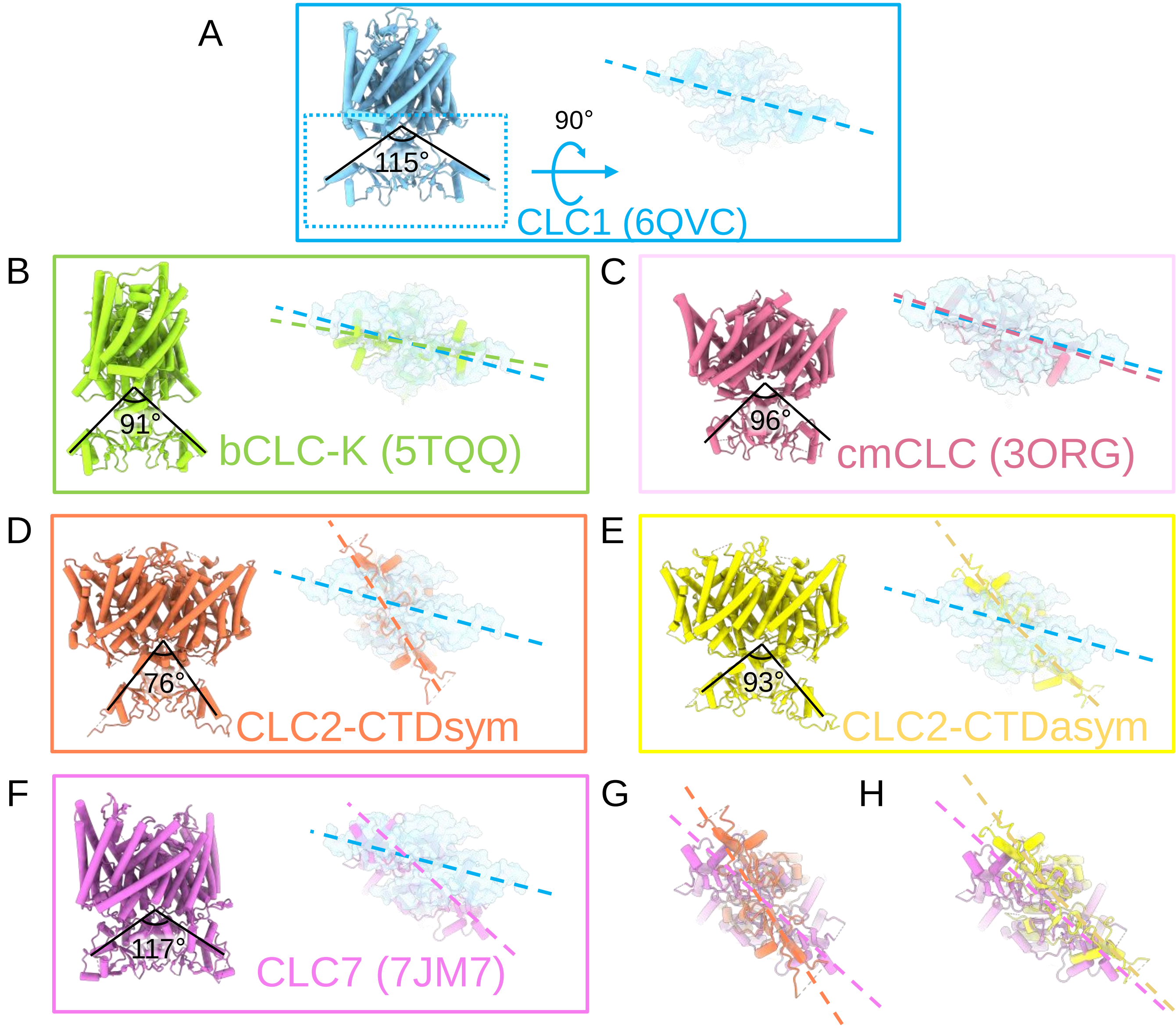

Figure 4 - figure supplement 4. Comparison of CTD orientation with respect to the TM domain. (**A**) The CLC-1 structure is shown in light blue. The angle of orientation for the CTDs was calculated using the far end residue of helix T (the second helix of first CBS domain) as the end point of rays and the center of the TM-CTD connecting plane as the vertex of the angle. On the right the CTD is shown in surface representation following rotation as indicated. Panels (**B**) – (**F**) show the CLC homolog indicated, with angles calculated as for CLC-1, and the rotated view of the CTD shown to compare the positioning of the CTDs relative to the membrane domains. The dashed lines indicate the long axes of the CTDs. In each panel, CLC-1 is shown in light blue as a reference for comparison. (**G**) and (**H**) overlays compare the CTD orientations in the CLC-2 structures to that in CLC-7. PDB IDs are shown in the parentheses. PDB IDs for CLC2-CTDsym and CLC2-CTD asym are 8TA4 and 8TA5.

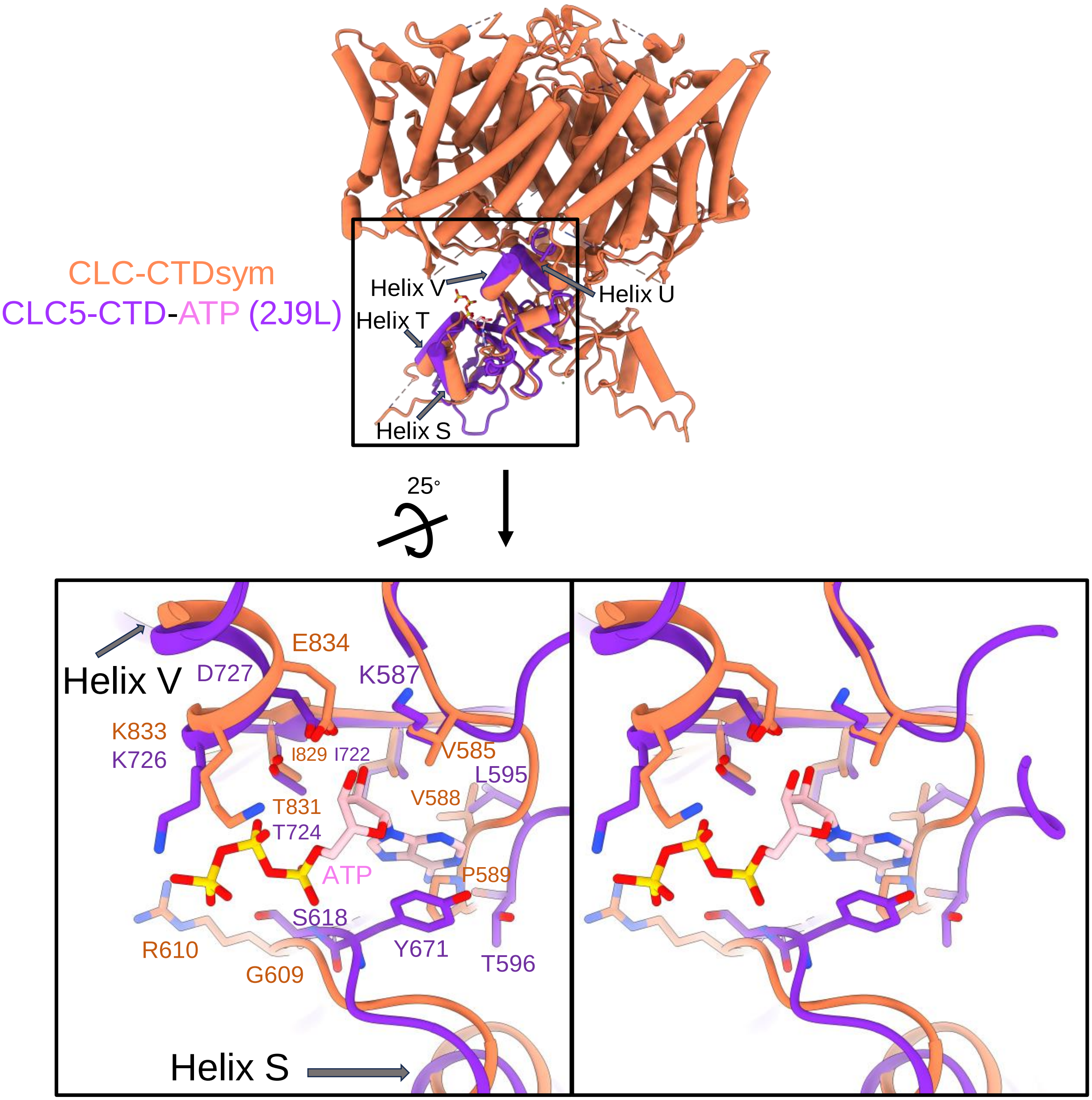

Figure 4 – figure supplement 5. ATP binding site comparison between CLC-2 and CLC-5. Top: Structural overlay of CLC2-CTDsym (in orange) and CLC5-CTD (in dark purple) with ATP (pink carbon atoms; CPK coloring on non-carbon atoms. PDB ID: 2J9L). Bottom: Zoomed in stereo view of ATP binding site in CLC5-CTD (dark purple) overlaid with the same region in CLC2-CTDsym (orange). Residue numbers are labeled in dark purple for CLC-5 and orange for CLC-2.

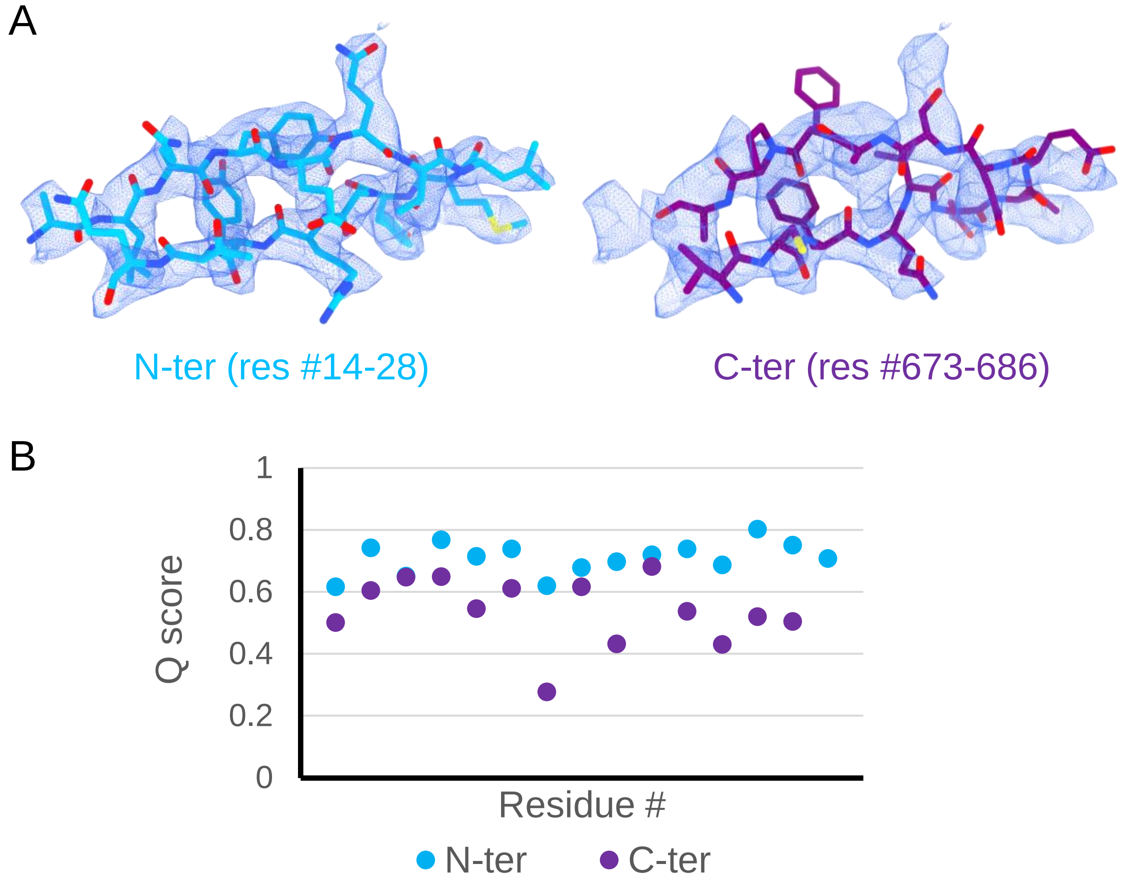

Figure 5 – figure supplement 1. Comparison of peptide candidates fitting into the hairpin density. (**A**) Best-fitting peptide candidate from the N-terminus (left shown in blue) and from the C-terminus (right shown in purple) fitted into cryoEM hairpin density (blue mesh). (**B**) Q-score plot of the two peptide candidates (residues 1-15 of the respective sequences). The Q-scores for the C-terminal peptide are generally lower than the Q-score expected for the corresponding resolution (0.65).

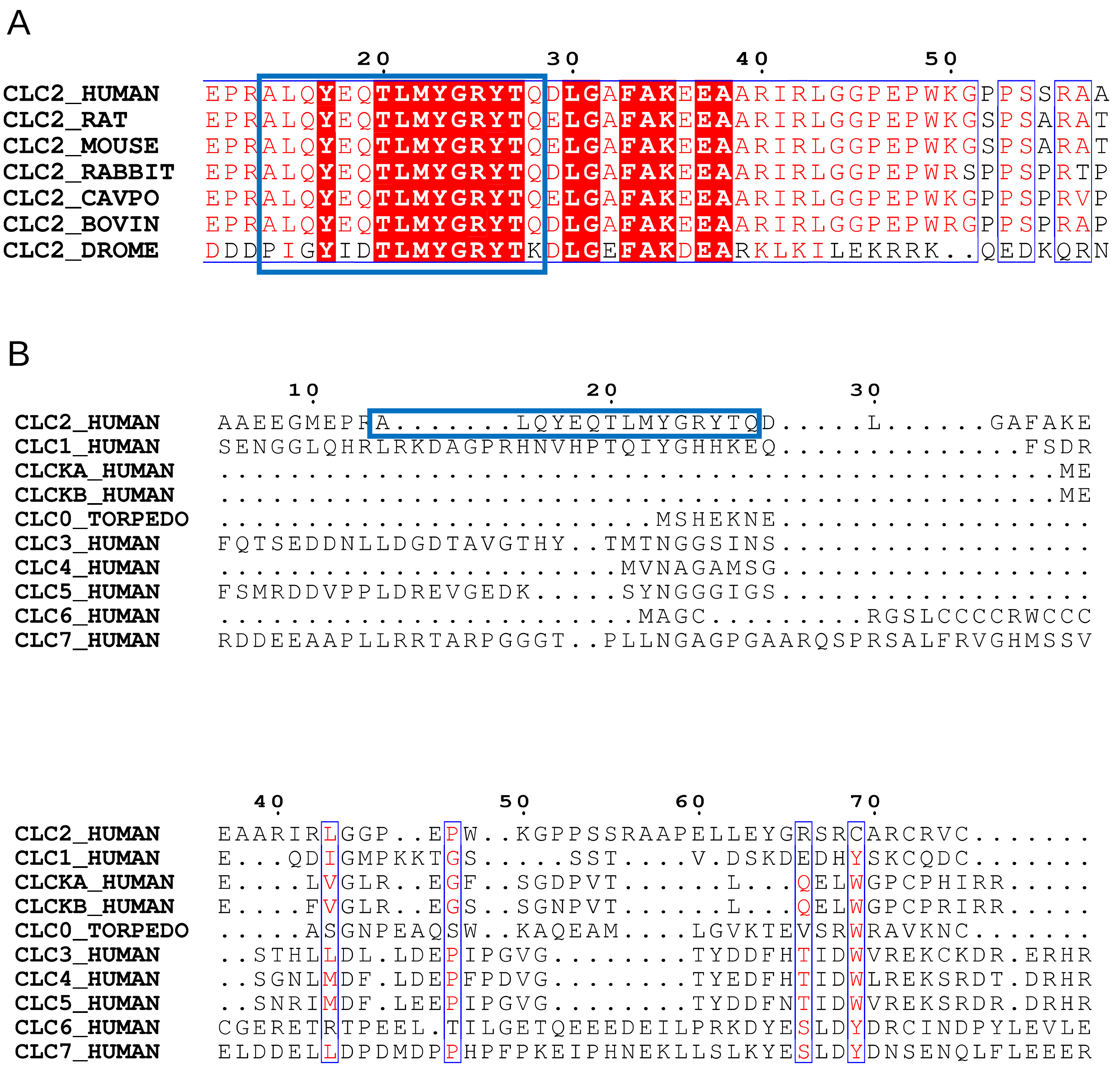

Figure 5 – figure supplement 2. N-terminal hairpin sequence is conserved in CLC-2, but not other CLCs. (**A**) Alignment of the N-terminal hairpin sequence (blue boxed) amongst different species. (**B**) Alignment of N-terminal hairpin sequence (blue boxed) amongst other human CLCs.

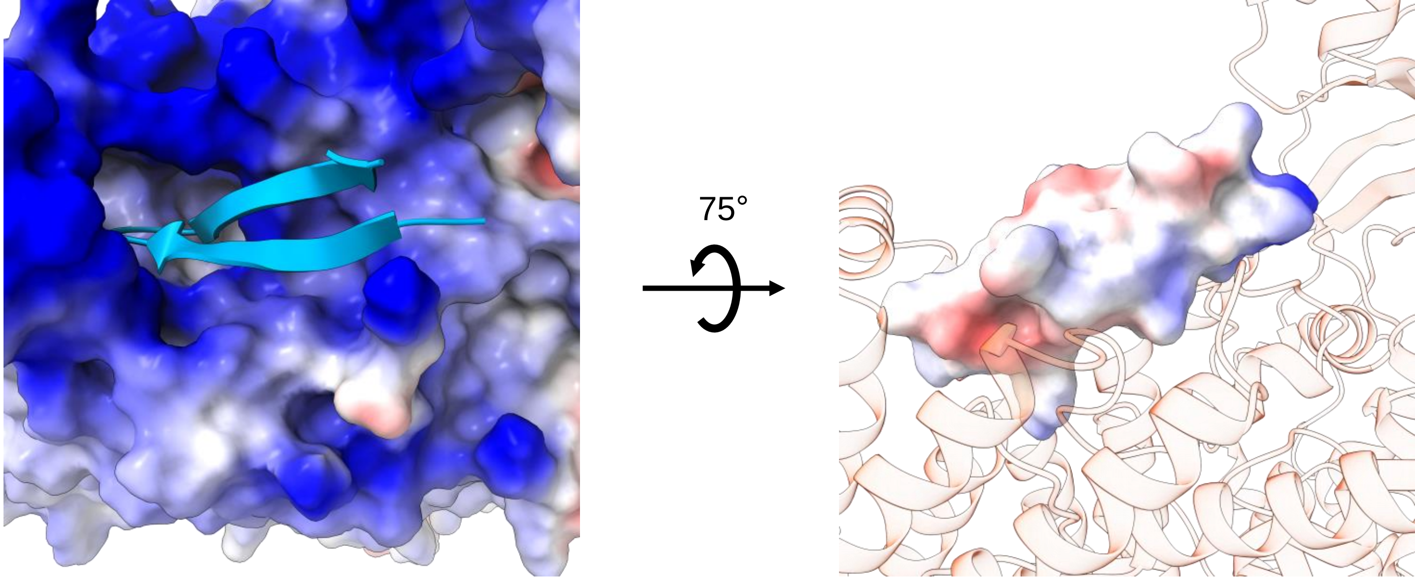

Figure 5 – figure supplement 3. Electrostatic map of the N-terminal hairpin blocking site. Left: view from the cytoplasm showing the electrostatic surface potential of the TM region (blue, positive; red, negative), with the hairpin shown in cartoon. Right: side view showing the electrostatic surface potential of the hairpin (blue, positive; red, negative) and the TM blocking site shown in transparent cartoon model.

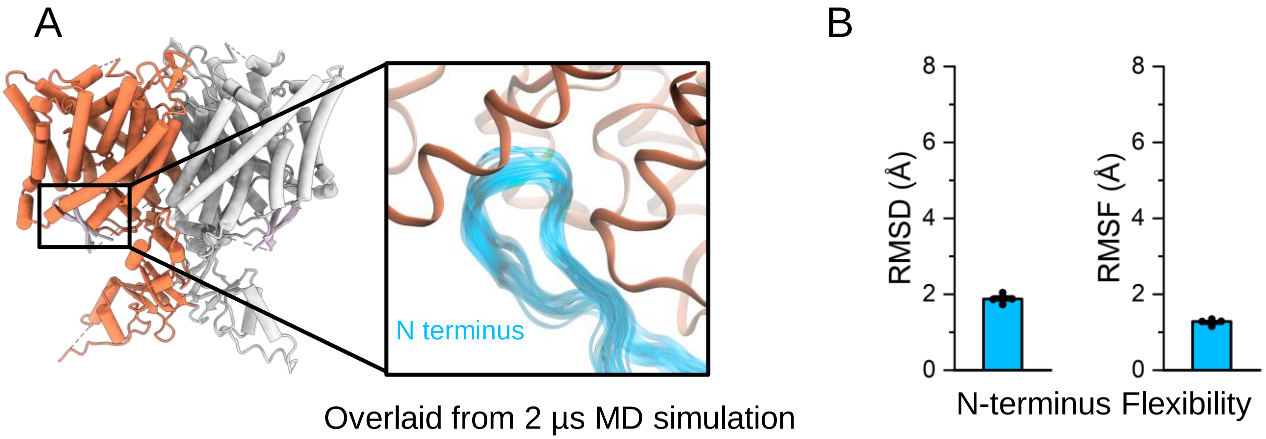

Figure 5 - figure supplement 4. MD simulations confirm the N-terminal hairpin structure is stable (**A**) In simulations, the N-terminus (blue) remains stably bound to the intracellular side of the transmembrane domain (orange). In the inset image, frames of the N-terminus taken every 200 ns from a representative simulation are overlaid. (**B**) The root-mean-square deviation (RMSD) and root-mean-square fluctuation (RMSF) of the N-terminus backbone were calculated after aligning frames on the transmembrane domains. Bars show the mean of five independent simulations, each 2.0 μs in length. Error bars are 68% CI (confidence interval of the mean).

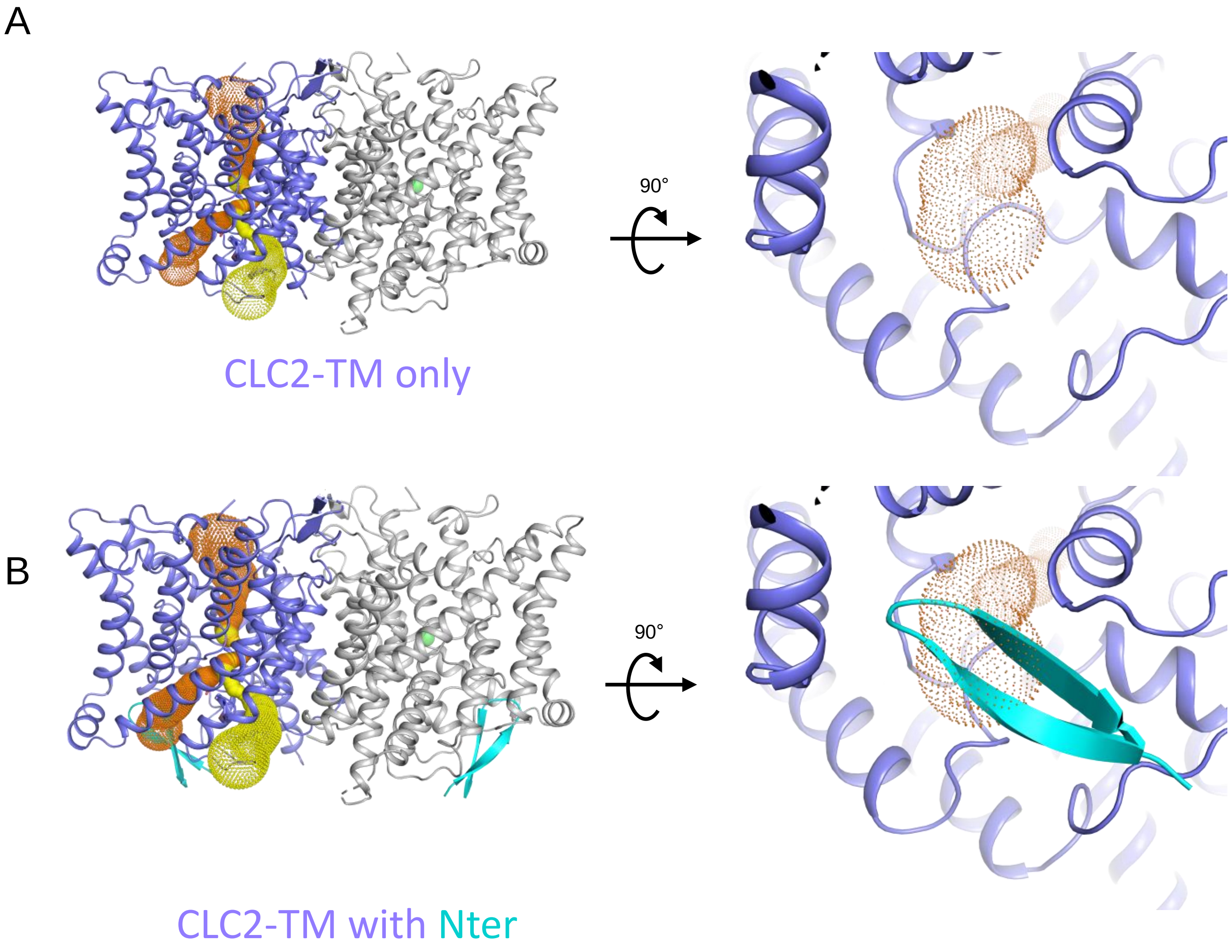

Figure 5 - figure supplement 5. The N-terminal hairpin blocks the primary Cl⁻ pore in CLC-2. (**A**) *Left:* Side view of CLC2-TM showing the caver-detected pore (primary in orange and secondary in yellow). *Right:* View of the primary pore from the intracellular side. (**B**) The N-terminal hairpin (blue) overlaid with CLC2-TM as shown in panel **A**.

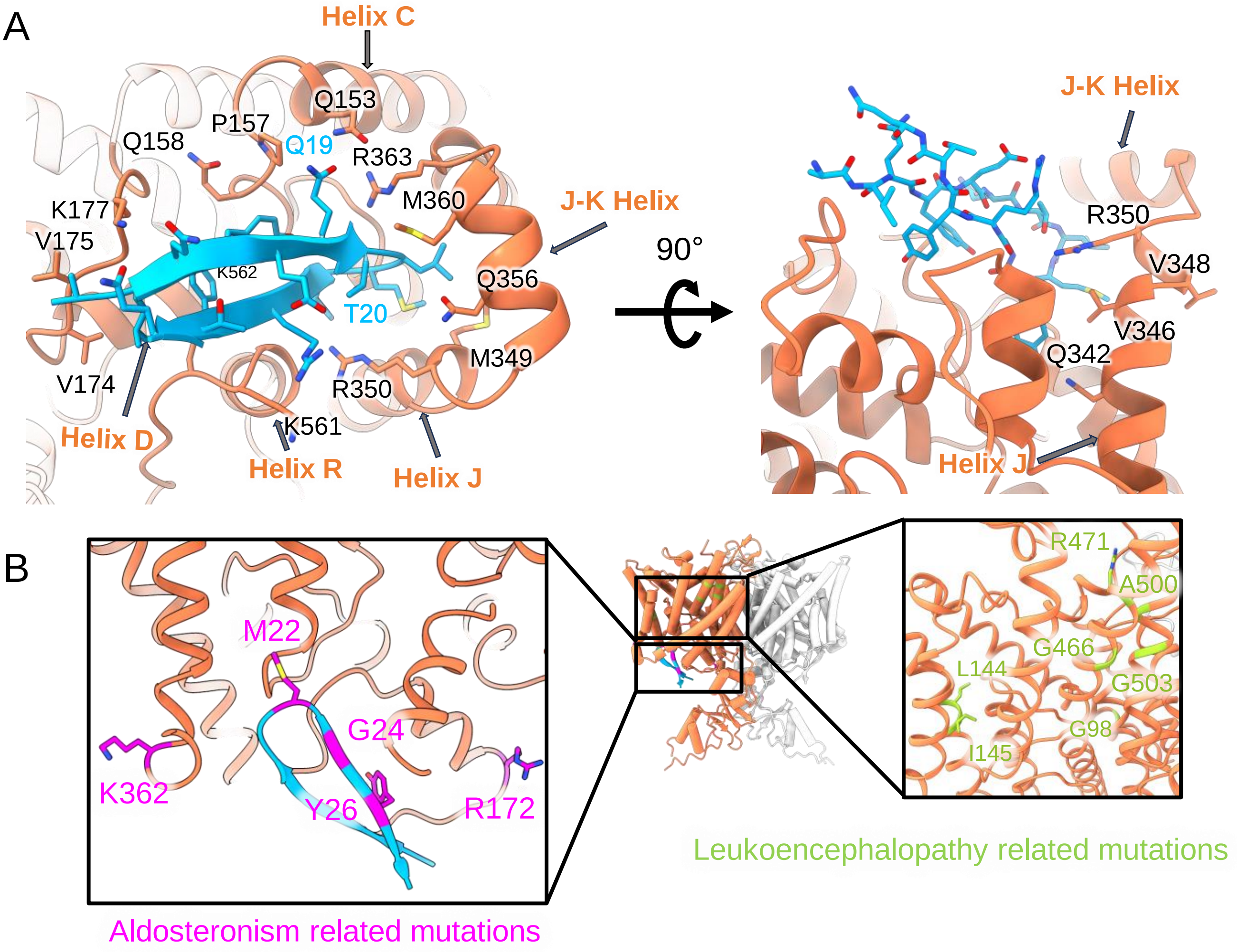

Figure 5 – figure supplement 6. Mapping mutant data onto the cryoEM structure. (A) Left: same as Figure 5C, showing hairpin interactions. Right: Rotated view highlighting ball "receptor" residues identified by Jordt and Jentsch, 1997. (**B**) Residues mutated in human Aldosteronism (Fernandes-Rosa et al., 2018, Scholl et al., 2018) and Leukoencephalopathy (Hector Gaitan-Penas., 2017) are shown on CLC2-CTDsym in magenta and green, respectively.

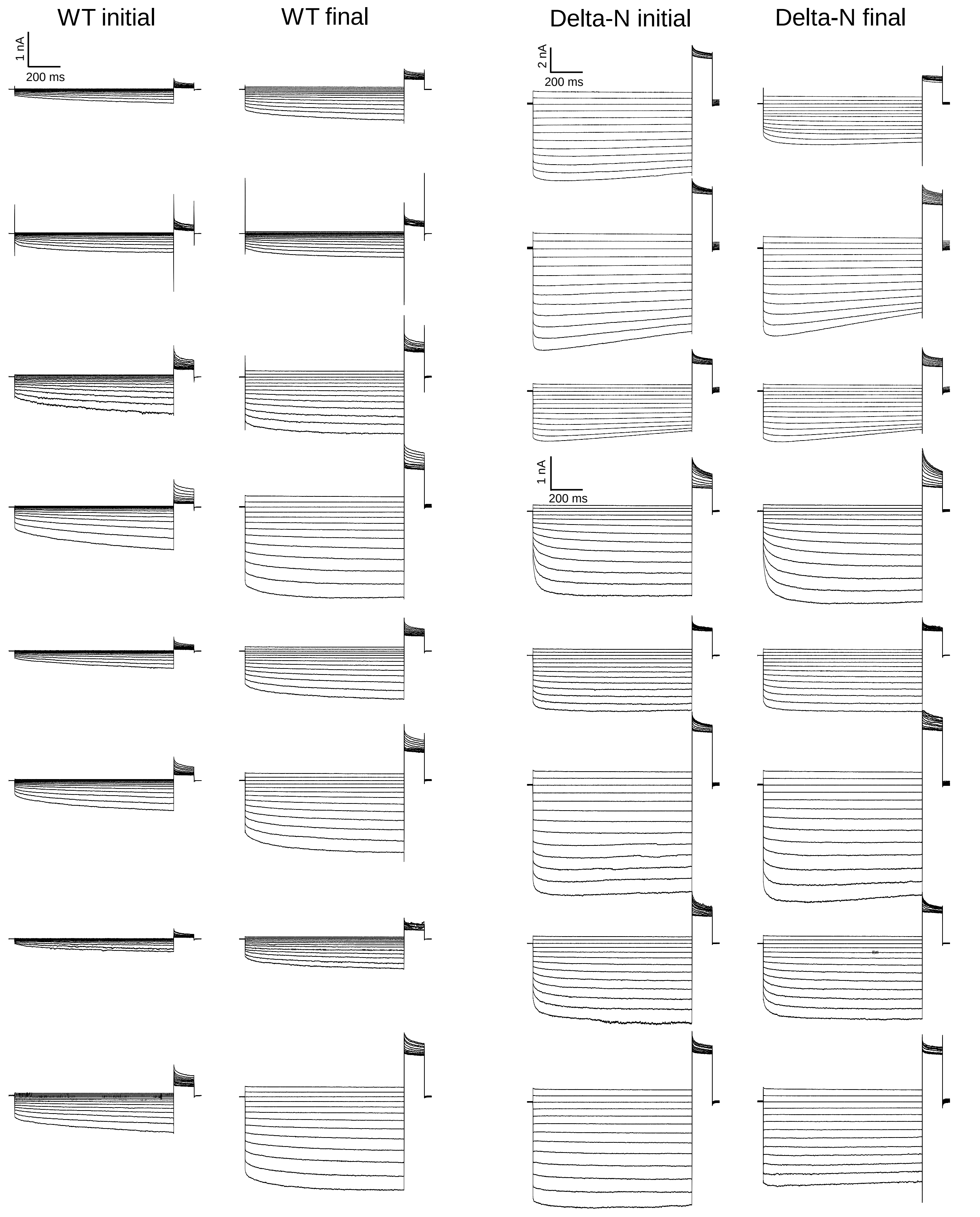

Figure 6 – figure supplement 1. Data traces from the CLC-2 run-up experiments summarized in Figure 6G. For Delta-N, separate scale bars indicate the currents for the top six and bottom 10 traces. AK-42 leak currents, not subtracted in these raw data traces, are summarized in Figure 6 – source data 1.

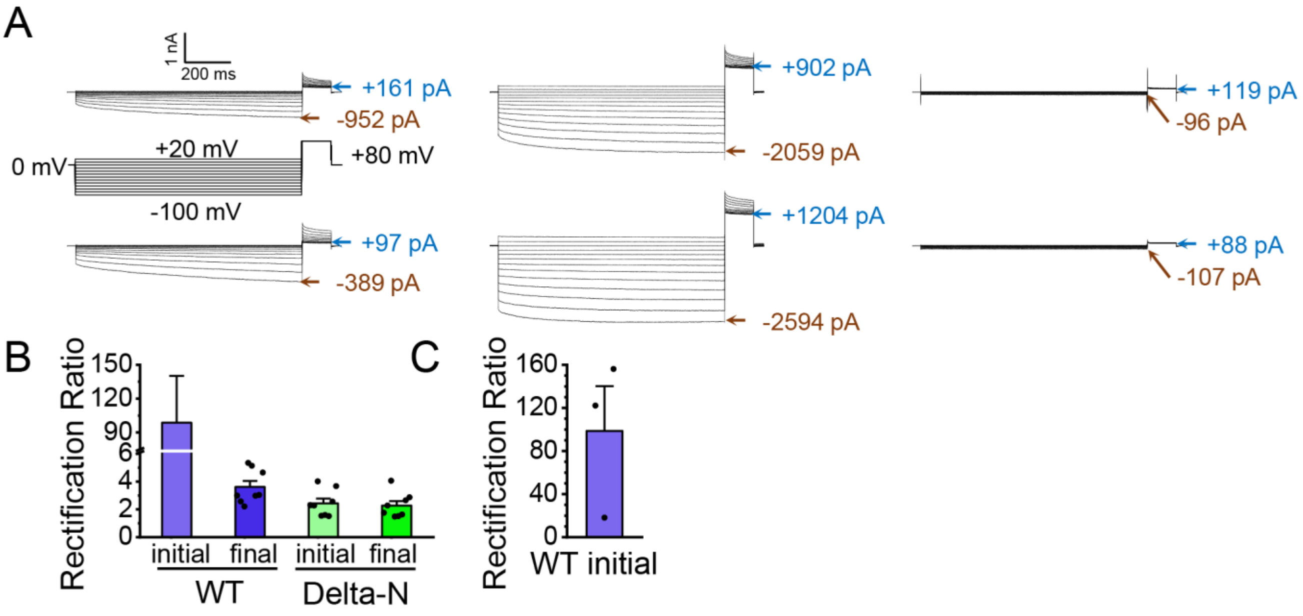

Figure 6 – figure supplement 2. CLC-2 current rectification. (**A**) Example traces from two experiments on WT CLC-2. Current values at end of the -100-mV test pulses and in the steady-state portion of the +80-mV tail pulse (following the +20-mV test pulse) are indicated. Robust AK-42 inhibition of the current at +80 mV indicates that CLC-2 is passing current at positive voltages. (**B**) Rectification ratios. Rectification was quantified by taking the ratio (absolute value) of the leak-subtracted currents at -100 mV and +80 mV. For WT initial currents, the low currents at +80 mV make this quantification challenging: for 5 out of 8 experiments on WT CLC-2, the AK-42 current at +80 mV was greater than the initial WT current at +80 mV, likely due to an increase in leak over the course of the experiment (~15 minutes of voltage pulsing). Therefore, the rectification calculated for the WT initial currents (99 ± 34, SEM n=3) has substantial uncertainty. That said, such high rectification is consistent with reports of CLC-2 rectification throughout the literature (Park et al., 1998; Arreola et al., 2002; Jentsch and Pusch, 2018). On the other hand, Delta-N currents display only mild rectification, which is straightforward to quantify given the high signal:noise (high AK-42-sensitive current relative to background), and which does not change in response to voltage pulsing. For “WT-final”, the rectification ratio similarly can be accurately quantified. Thus, while the high uncertainty and variance in estimating WT initial rectification precludes quantitative statistical comparisons, these data strongly suggest that WT CLC-2 current rectification decreases in parallel to current run-up. (**C**) WT initial rectification ratio from panel B replotted to show the individual data points from the 3 experiments in which AK-42 current at +80 mV was less than the initial WT current at +80 mV. Data for all experiments are summarized in Figure 6 – source data 1.

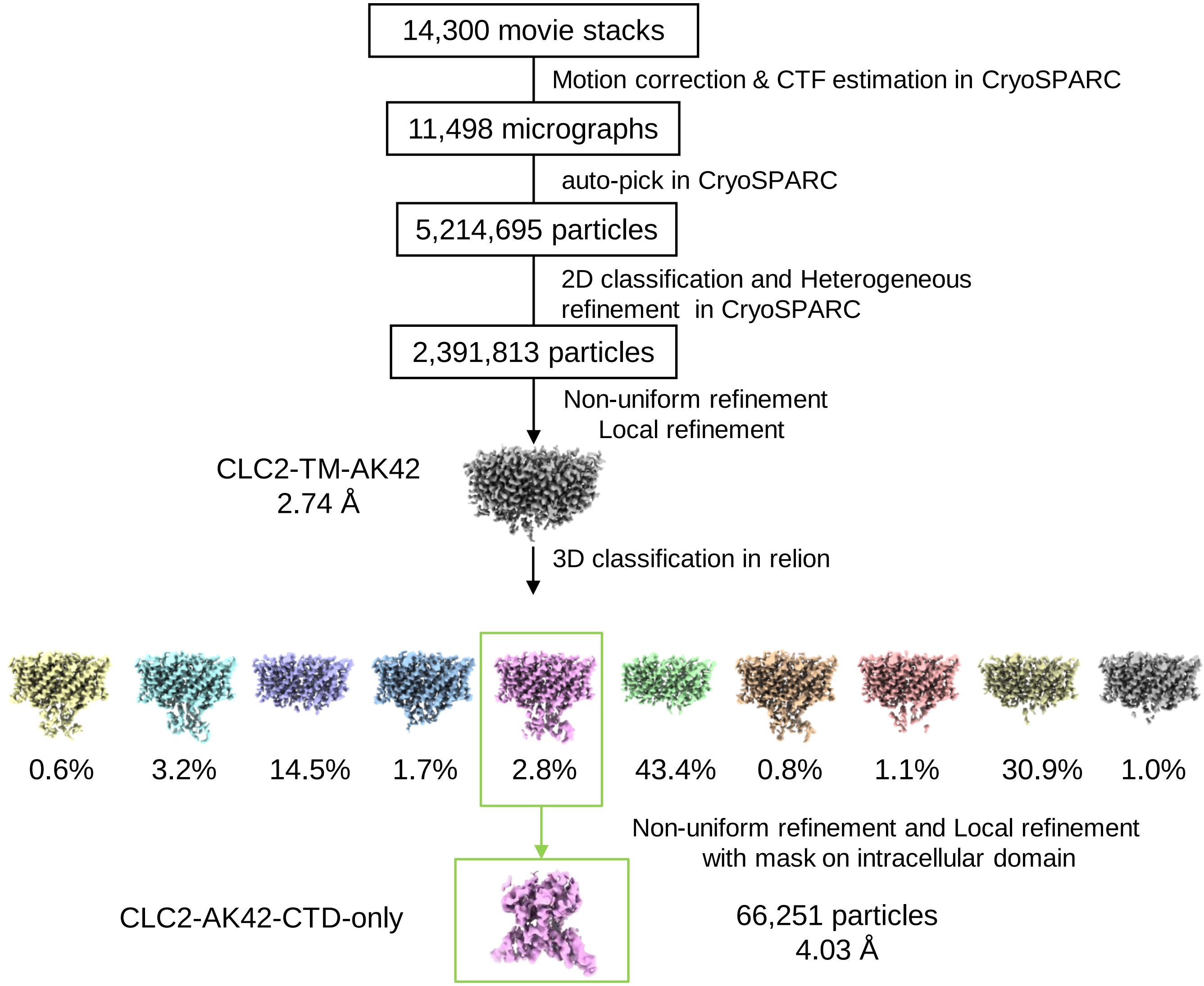

Figure 7 - figure supplement 1. cryoEM workflow of the CLC2-TM-AK42 single-particle cryo-EM data processing. A total of 14,300 movie stacks were collected on a 300 kV Titan Krios cryo-electron microscope. cryoSPRAC was used for 2D classification and CLC2-TM-AK42 density map was obtained after 2D classification. Relion was used for 3D classification.

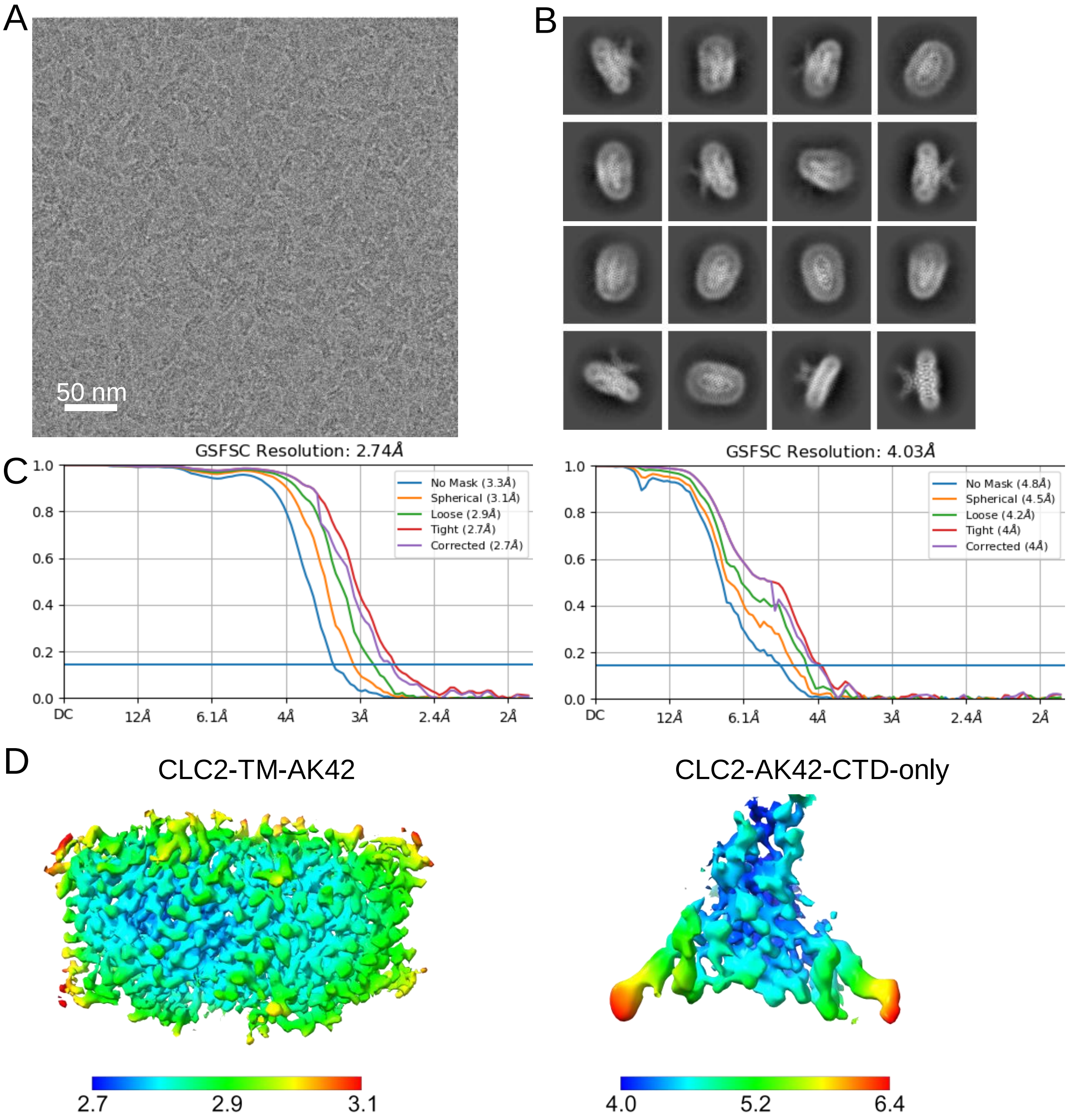

Figure 7 - figure supplement 2. Micrograph and 2D classes and structure validation of CLC2-AK42. (A) Representative motion-corrected cryo-EM micrograph. (B) 2D class averages. (C) Gold standard FSC plots calculated in cryoSPARC for CLC2-TM-AK42 (left) and CLC2-AK42-CTD-only (right). (D) Local resolution of the cryo-EM map for CLC2-TM-AK42 (left) and CLC2-AK42-CTD-only (right)

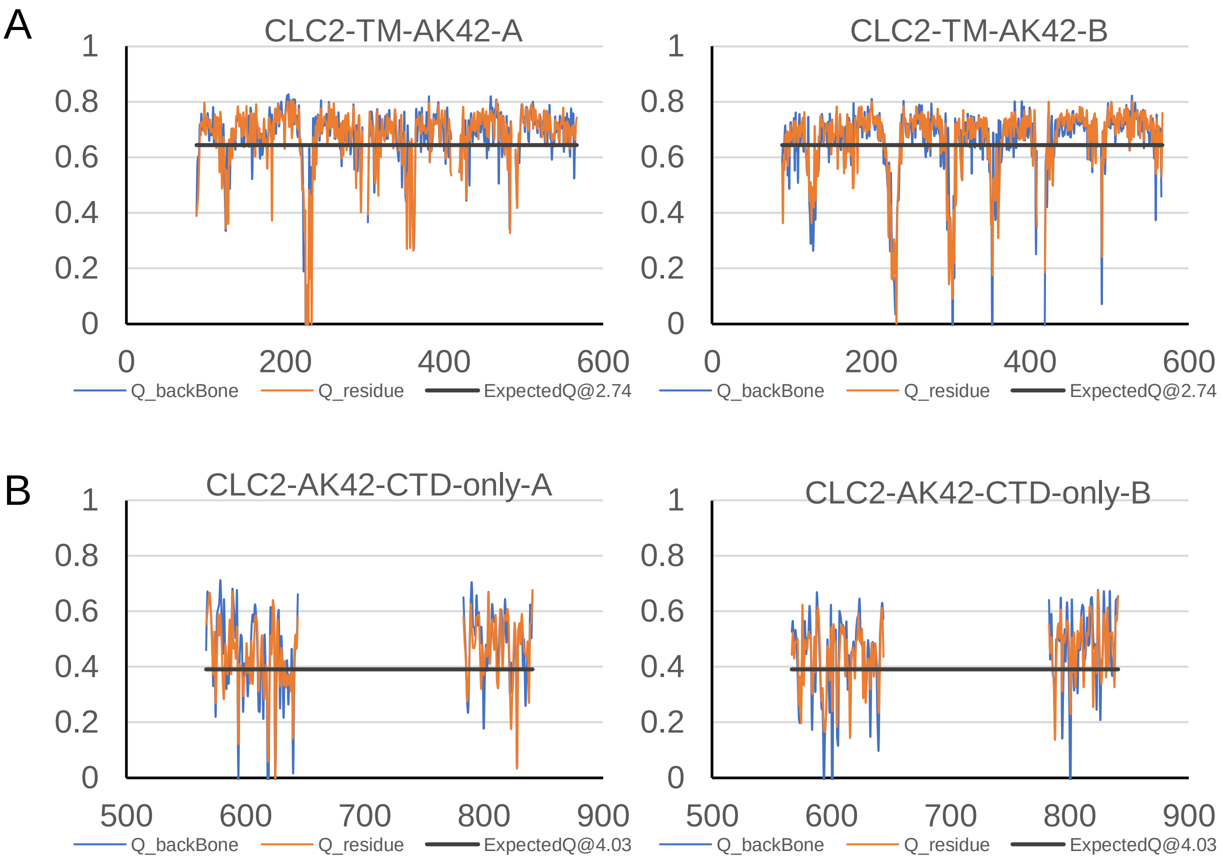

Figure 7 - figure supplement 3. Model validation using Q-scores. (**A**) Q-scores of CLC2-TM-AK42 for subunit A (left) and subunit B (right). (**B**) Q-scores of CLC2-AK42-CTD-only for subunit A (left) and subunit B (right). The black line represents the expected Q-score at respective resolution based on the correlation between Q-scores and map resolution.

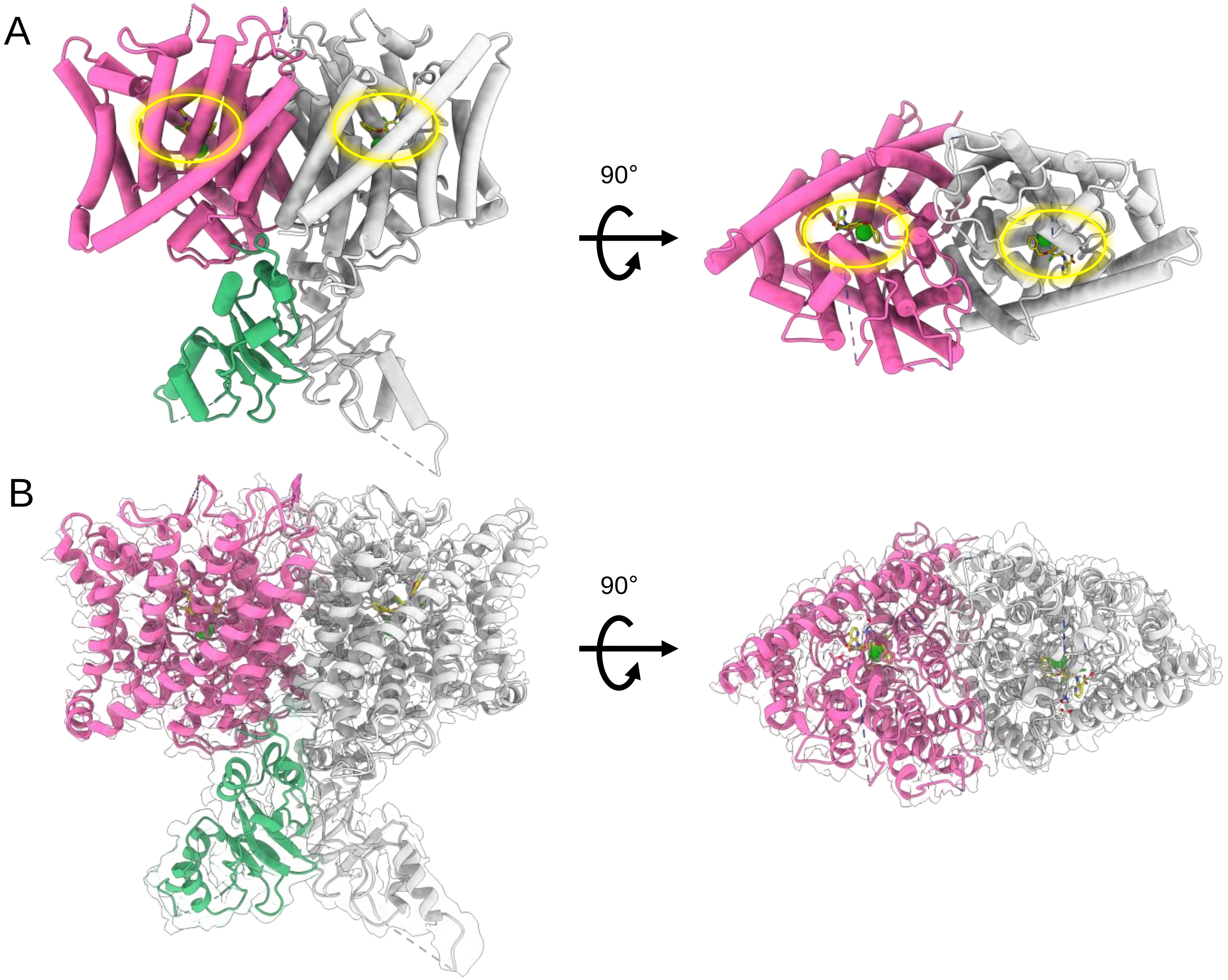

Figure 7 - figure supplement 4. cryoEM map and structure of CLC2-AK42. (**A**) cryoEM model of CLC2-TM-AK42 with docked CTD. The transmembrane region is shown in hot pink and gray. The CTD is shown in green and gray. AK-42 is shown in yellow and highlighted by yellow circles. Chloride ions are shown in green. (**B**) cryoEM model overlay with density map of CLC2-AK42. Contour level: 0.83, 6.8 σ (TM region); 0.35, 14 σ (CTD).

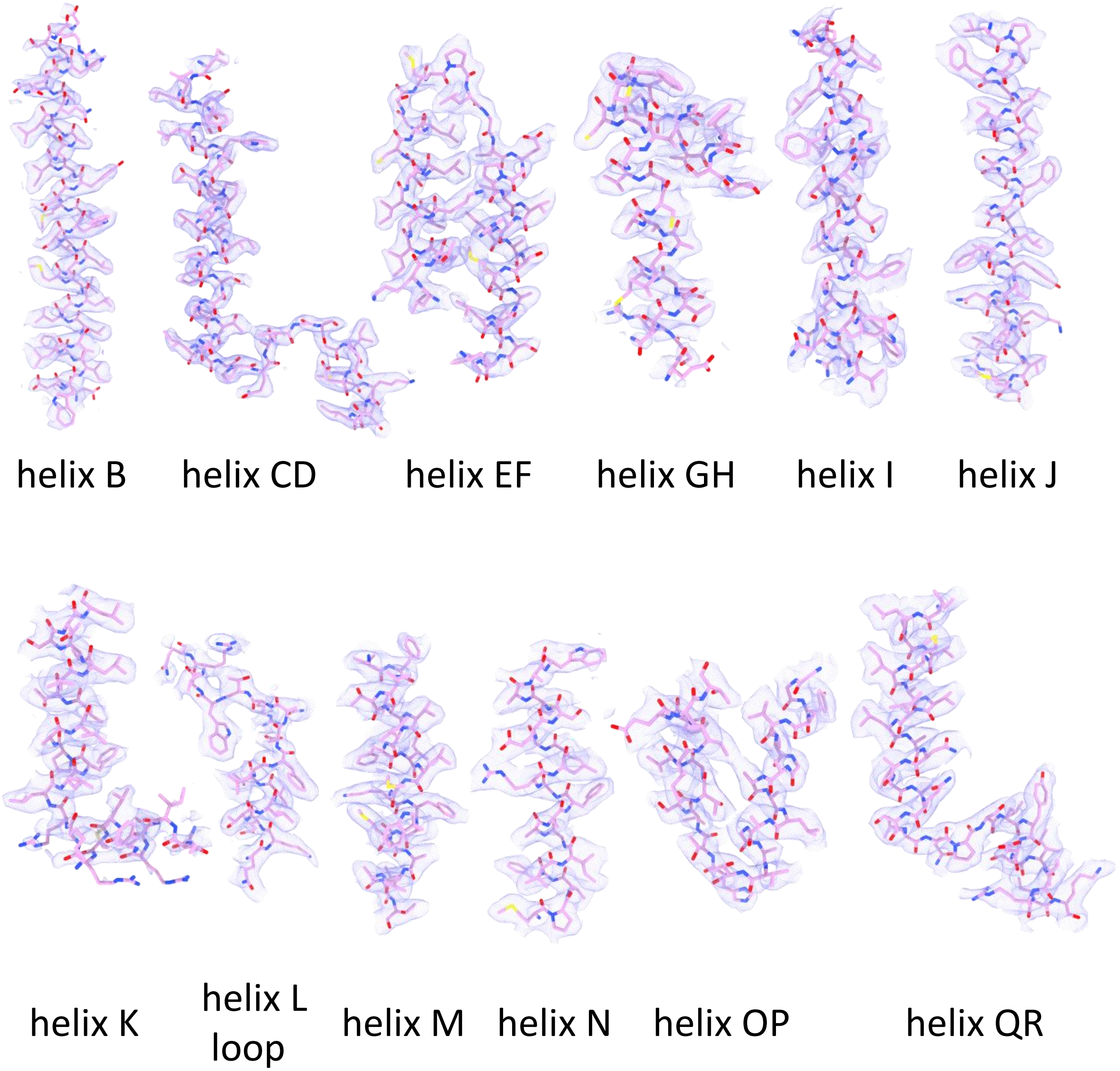

Figure 7 - figure supplement 5. Helix map of CLC2-TM-AK42. cryo-EM densities and model of CLC2-AK42 transmembrane helices (contour level: 0.52, 3.4 σ).

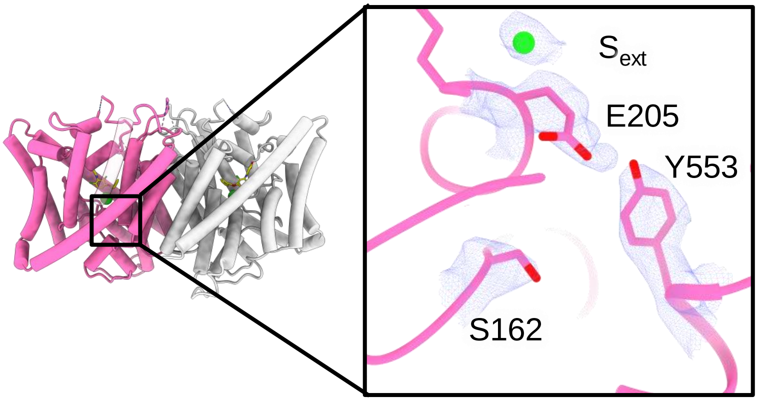

Figure 7 – figure supplement 6. Density overlay at key Cl⁻-pathway residues. Zoomed-in view highlighting residues S162, E205, Y553, and the bound chloride ion, with cryoEM density overlay (contour level: 1.4, 9.0 σ).

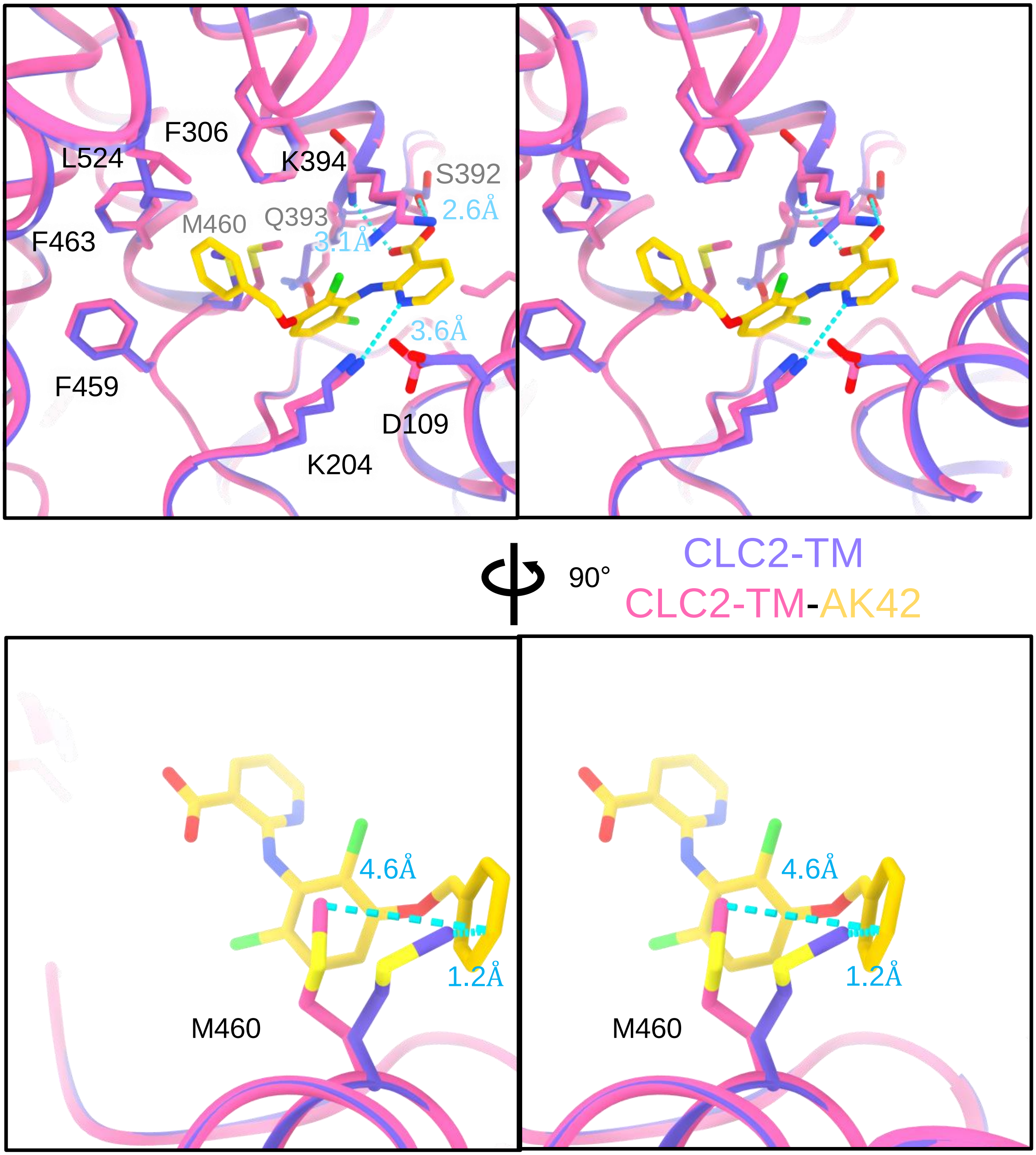

Figure 7 - figure supplement 7. AK-42 binding site comparison between CLC2-TM and CLC2-TM-AK42. Top: Stereo view of structural overlay at the AK-42 binding site. Hydrogen bonds between AK-42 and residues K204,S392,K394 are indicated by blue dashed lines. Bottom: A view highlighting the movement of residue M460 to make space for AK-42.

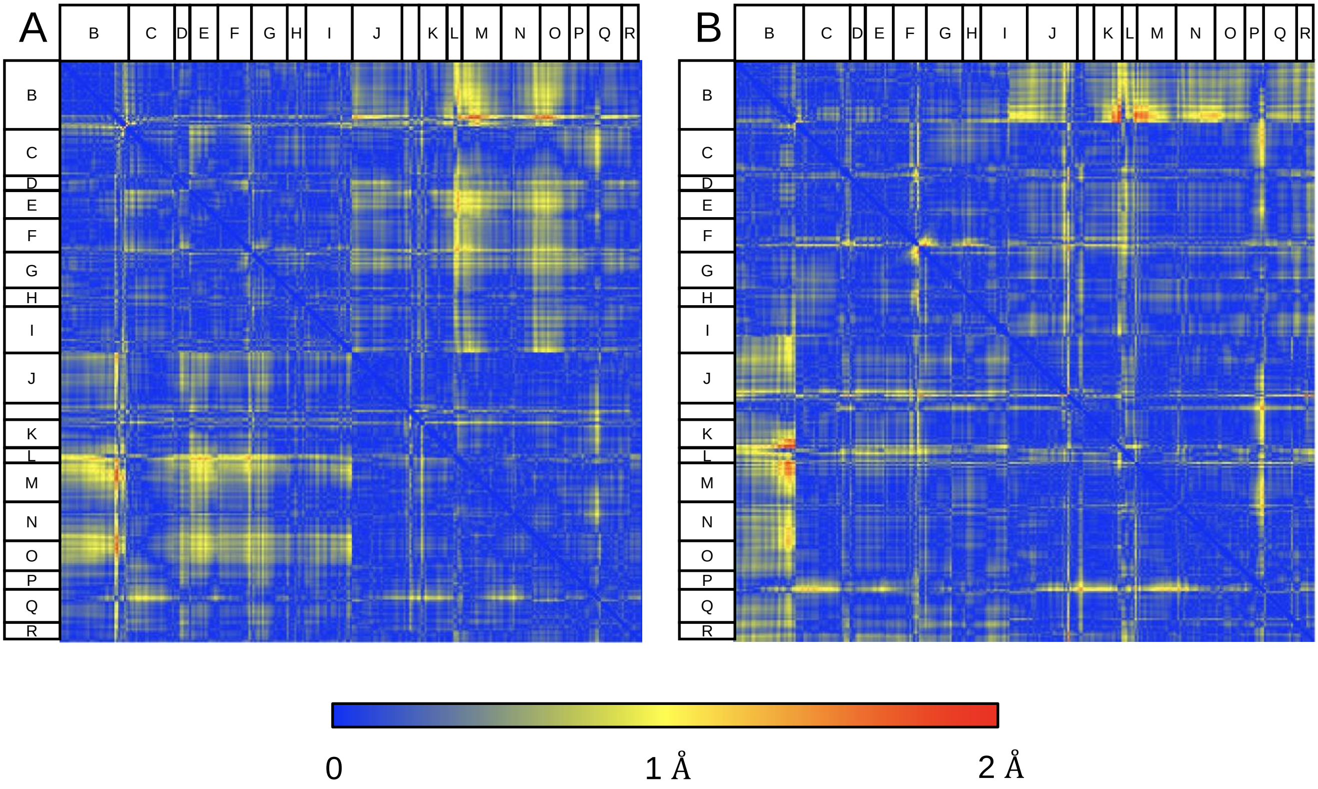

Figure 7 - figure supplement 8. Structural comparison of CLC2-TM and CLC2-TM-AK42. Difference distance matrices comparing Cα residues on TM helices between CLC2-TM and CLC2-TM-AK42 in subunits A (**A**) or B (**B**). Residues in loops connecting helices, where there is low confidence in the model building (Q score lower than the expected Q score of 0.65), were omitted from the matrices.

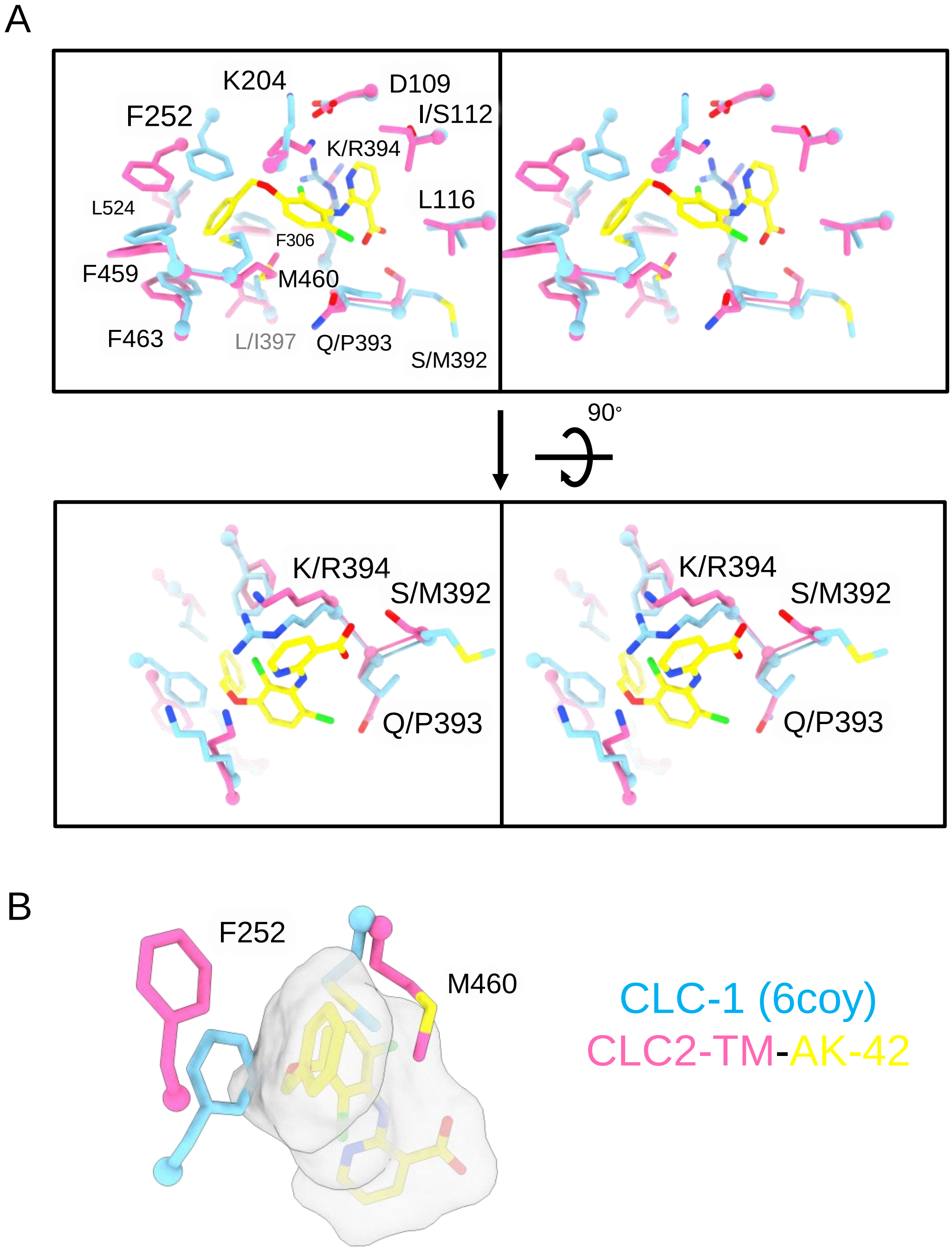

Figure 7 - figure supplement 9 AK-42 binding site comparison between CLC-2 and CLC-1. (**A**) Top: Stereo view of the AK-42 binding site in CLC2-TM-AK42 (hot pink) overlaid with the same region in CLC-1 (blue, PDB:6coy). Residue numbers correspond to those of CLC-2. The five residues that differ between CLC-2 and CLC-1 are: I112 (S139), S392 (M419), Q393 (P420), K394 (R421), and L397 (I424). Bottom: Rotated (stereo) view of AK-42 binding site to compare residues S392, Q393 and K394 in CLC-2 versus M419, P420 and R421 in CLC-1. These three were calculated to contribute most significantly to the AK-42 binding energy (Ma, 2023). (**B**) Residues in CLC-1 predicted to clash with AK-42. AK-42 is shown in yellow sticks and white surface. F252 and M460 in CLC-2 correspond to F279 and M485 in CLC-1.
